# A refined model of claudin-15 tight junction paracellular architecture by molecular dynamics simulations

**DOI:** 10.1371/journal.pone.0184190

**Published:** 2017-09-01

**Authors:** Giulio Alberini, Fabio Benfenati, Luca Maragliano

**Affiliations:** 1 Center for Synaptic Neuroscience & Technology (NSYN@UniGe), Istituto Italiano di Tecnologia, Largo Rosanna Benzi, 10, 16132, Genova, Italy; 2 Department of Experimental Medicine, Università degli Studi di Genova, Viale Benedetto XV, 3, 16132, Genova, Italy; University of Minnesota Twin Cities, UNITED STATES

## Abstract

Tight-junctions between epithelial cells of biological barriers are specialized molecular structures that regulate the flux of solutes across the barrier, parallel to cell walls. The tight-junction backbone is made of strands of transmembrane proteins from the claudin family, but the molecular mechanism of its function is still not completely understood. Recently, the crystal structure of a mammalian claudin-15 was reported, displaying for the first time the detailed features of transmembrane and extracellular domains. Successively, a structural model of claudin-15-based paracellular channels has been proposed, suggesting a putative assembly that illustrates how claudins associate in the same cell (via *cis* interactions) and across adjacent cells (via *trans* interactions). Although very promising, the model offers only a static conformation, with residues missing in the most important extracellular regions and potential steric clashes. Here we present detailed atomic models of paracellular single and double pore architectures, obtained from the putative assembly and refined via structural modeling and all-atom molecular dynamics simulations in double membrane bilayer and water environment. Our results show an overall stable configuration of the complex with a fluctuating pore size. Extracellular residue loops in *trans* interaction are able to form stable contacts and regulate the size of the pore, which displays a stationary radius of 2.5–3.0 Å at the narrowest region. The side-by-side interactions of the *cis* configuration are preserved via stable hydrogen bonds, already predicted by cysteine crosslinking experiments. Overall, this work introduces an improved version of the claudin-15-based paracellular channel model that strengthens its validity and that can be used in further computational studies to understand the structural features of tight-junctions regulation.

## Introduction

Biological barriers such as the blood-brain, renal or intestinal barriers are highly complex structures that perform the fundamental task of maintaining stable physical and chemical conditions of the compartments they separate. They are composed of closely joined epithelial cells whose lateral membranes are circumscribed by regions of narrow space named tight-junctions (TJs), comprising several proteins [1, 2]. TJs form strands that act as barriers between adjoining cells, but they also contain channels that regulate solute flow through paracellular spaces [3–6]. In contrast to transcellular diffusion, which involves primary (ATP-based) and secondary (electrochemical) active transport across the cell membrane together with diffusion through ion channels, paracellular transport occurs passively along a concentration gradient and is regulated by the TJ proteins [7–9]. While the mechanisms underlying transcellular permeation are fairly understood, the investigation of paracellular pathways has been hampered by the lack of structural information. This leaves major challenges such as the design of TJ penetrating drugs and nanomaterials unresolved [10].

TJs are predominantly formed by claudins, a family of proteins made of four *α*-helices (named TM1 to TM4) and two extracellual loops (ECL1 and ECL2), which show tissue-specific localization and function [11–15]. While TJs also contain other proteins, claudins alone can reconstitute TJ-like strands in plasma membranes [16]. It is now accepted that paracellular pores are formed by *trans* (intercellular) association of claudins. However, although previous reports have demonstrated that claudin extracellular domains are involved in cell adhesion and solute selectivity [17–19], the molecular mechanisms are still poorly understood. Recently, the crystal structure of mouse claudin-15 (Cldn15) was resolved (PDB ID: 4P79 [20]), providing a breakthrough in TJ research. Two other crystal structures of claudins were produced afterwards in complex with enterotoxins (mCldn19 [21] and hCldn4 [22]), providing clues about TJ disruption.

**Fig 1:**
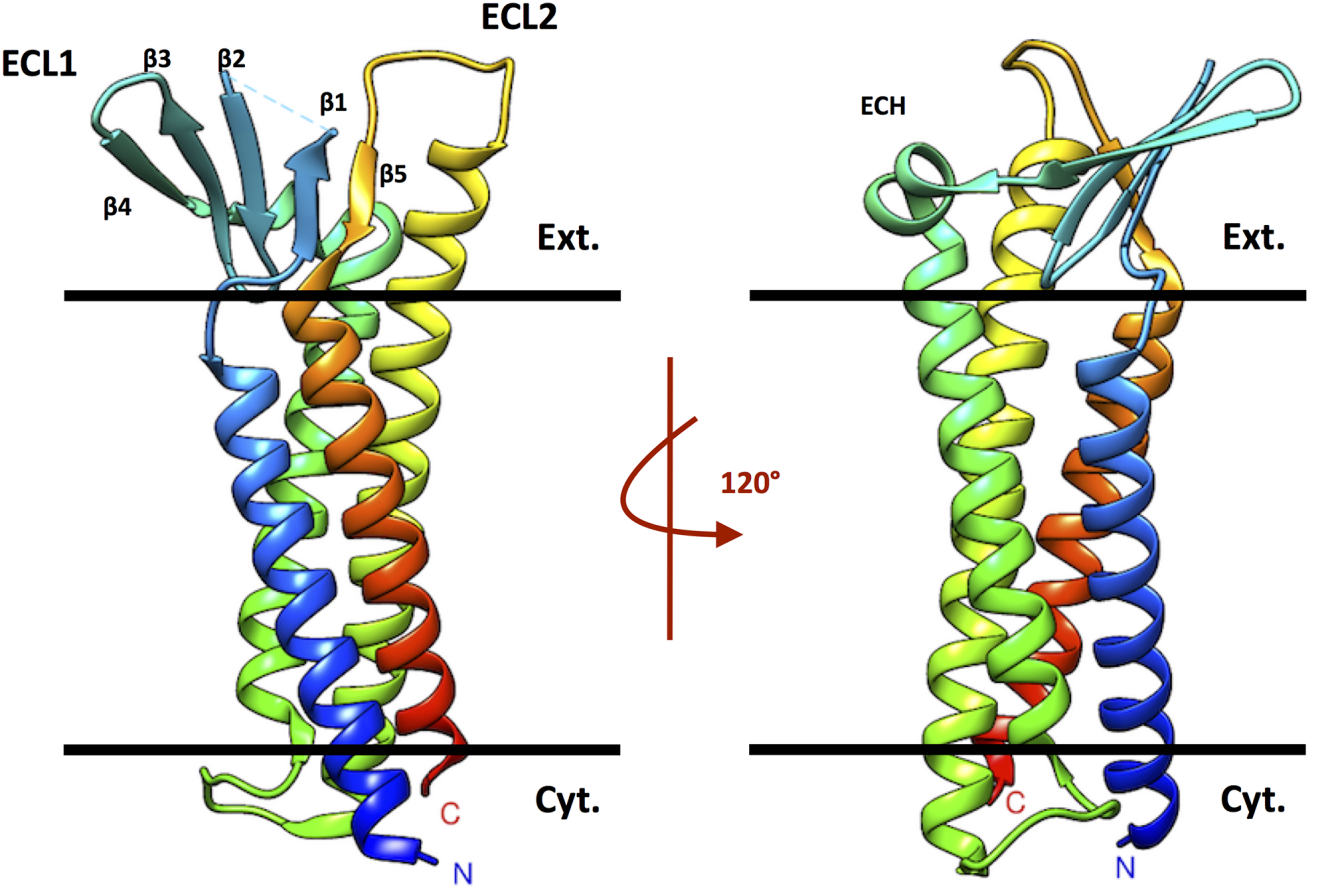
Crystal structure of Cldn15. Ribbon representation of the Cldn15 crystal structure (PDB code 4P79). The black bars indicate the membrane boundaries with the extracellular (Ext.) and cytosolic space (Cyt.).

Highly expressed in the intestine, Cldn15 protomers form selective pores that are channels for Na^+^ ions and highly resistant barriers to Cl^−^. The structure of the Cldn15 protomer displays a characteristic *β*-sheet fold, formed by two extracellular loops, which comprises five *β* strands (named *β*1 to *β*5), four (*β*1 to *β*4) in the longer ECL1 and one (*β*5) at the end of the shorter ECL2 (Fig 1). At the C terminus of ECL1, the *β*4 strand connects to a short extracellular helix (ECH) that links to the TM2 domain. Strikingly, the loop region between *β*1 and *β*2 (residues 34 to 41) is missing in the crystal structure. The monomeric structure of Cldn15 has been employed in coarse-grained computational studies [23], and as a template to build structural models of several other claudins [24, 25].

In the crystal lattice, Cldn15 protomers assemble in a linear polymer via specific interactions mediated by residues in the extracellular domains (S1 Fig). In particular, residue M68 of the ECH region of one protomer interacts with residues F146, F147, L158 in TM3 and ECL2 of an adjacent protomer. However, this linear arrangement is not considered sufficient to explain how claudins arrange themselves in TJ strands.

**Fig 2:**
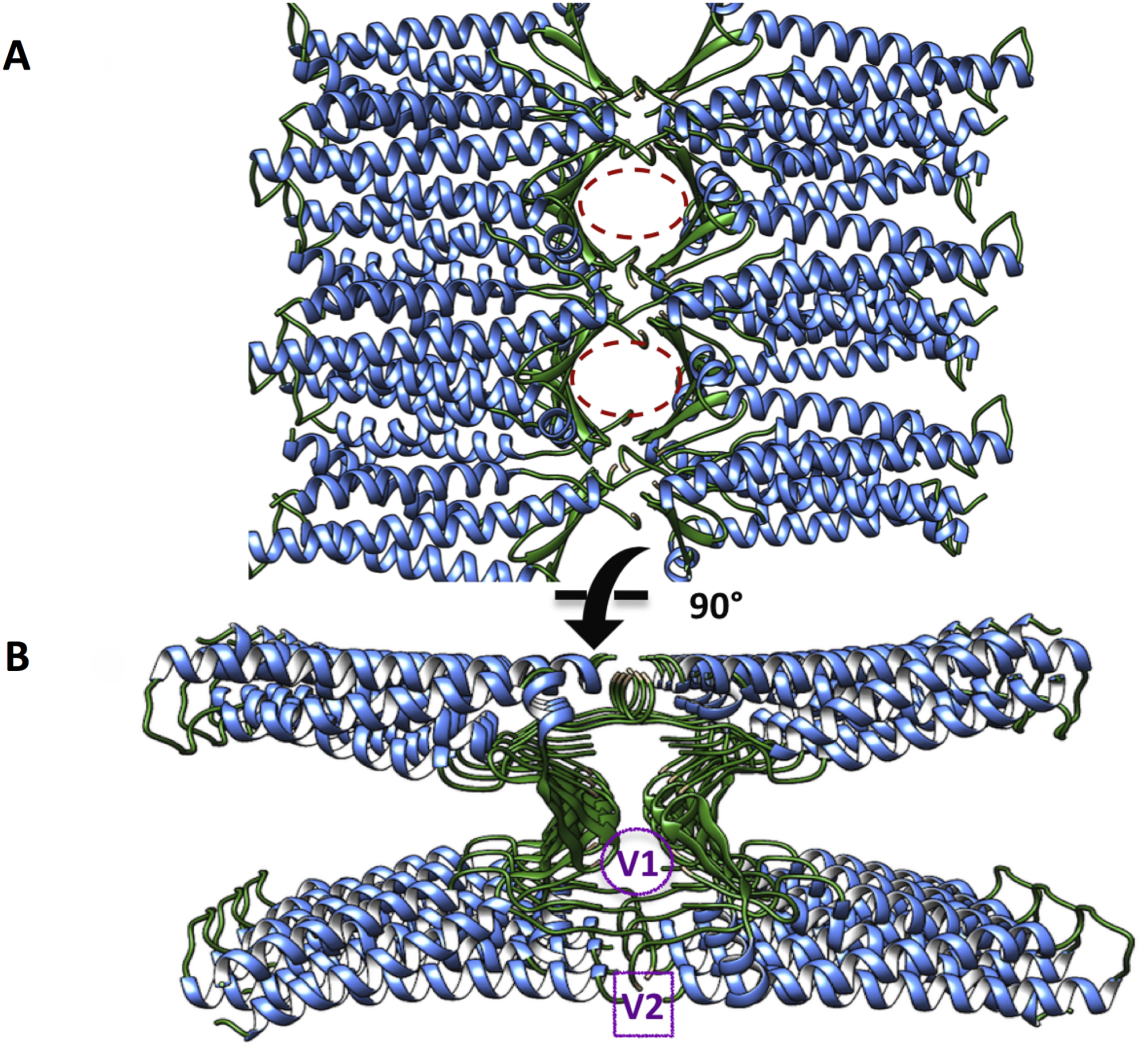
Model of paracellular TJ channels proposed by Suzuki et al. The model of claudin-based paracellular channels proposed by Suzuki et al., represented as ribbons and viewed from the apical (**A**) and the lateral side (**B**). The pore regions are marked with purple circles, and the V1 and V2 regions (see text) are indicated.

Soon after the publication of the Cldn15 monomer crystal, a model of paracellular channels was proposed using the same structure [26] (Fig 2). This configuration combines two distinct *cis* interfaces, renamed linear and face-to-face in [27]. The first is the same observed in the crystal lattice described above, while the second is formed through interactions between the edges of *β*4-strands of ECL1 of side-by-side protomers (S2 Fig). The introduction of this arrangement is supported by cysteine crosslinking data obtained from mutants of Cldn15 and another pore-forming claudin (Cldn2) [27]. Most importantly, the model suggests the formation of *β*-barrel-like channels with diameter smaller than 10 Å by the association of antiparallel claudin double rows in adjacent membranes. The assembly relies on a putative *trans*-interaction between the two *cis* strands mediated by the interaction of regions in ECL1 and ECL2, named V1 (residues 31 to 46) and V2 (residues 150 to 154), respectively, that are poorly conserved among different claudins.

The suggested arrangement of protomers is consistent with freeze fracture electron microscopy (EM) images of polymeric TJ strands [26], and displays pore facing (D55) and pore lining (D64) negatively charged residues whose mutation was shown to reverse claudin selectivity for cations [28]. Nevertheless, its general validity is still debated [27]. First of all, barrier-forming claudins such as Cldn3 and Cldn5 might arrange differently from channel-forming ones, consistent with their demonstrated role against passage of ions and small molecules. Additionally, the Cldn15 protomers used to assemble the model show an incomplete topology, with nine residues missing in ECL1 (eight of which are absent also in the crystal structure), and two in ECL2 (S3 Fig). It has been suggested [27] that the assembly implies very tight head-to-head packing in the regions of missing residues, which might cause excessive steric disturbance once these are added. Interestingly however, computational studies based on coarse-grained models [24] demonstrated that for a different claudin (Cldn5), whose structure was modeled by homology with Cldn15, a *cis* arrangement similar to that of Suzuki et al. is among those that are spontaneously formed by monomers. Morover, by docking these Cldn5 dimers [25], it was possible to reconstruct a single-pore structure similar to the Cldn15 model of Suzuki et al.

Overall, the structural model proposed by Suzuki et al. provides an extremely valuable starting hypothesis for the molecular architecture of claudin-based TJ pores, which demands further testing in order to assess its validity.

In this work, we explore via all-atom molecular dynamics (MD) simulations different paracellular pore structures made of Cldn15 monomers built starting from the model by Suzuki et al., with the aim of assessing its structural stability and conformational properties. Our main result is a refined model of a single paracellular pore made of four *trans*-interacting monomers embedded in a double lipid bilayer, obtained via MD simulations lasting about 260 ns. The tetrameric assembly is based on the *trans* interaction of two face-to-face *cis* dimers, and it is the minimal structure consistent with the model of Suzuki et al. that is sufficient to reproduce an individual paracellular pore. It displays inter-monomer residue-residue interactions (both *cis* and *trans*) that are described in the literature as relevant for maintaining TJ integrity and function. Over the simulated trajectory, the structure shows global structural stability, preserving the relevant interactions between monomers. Fluctuations in the extracellular loops affect the size of the pore, whose maximal constriction is mostly stationary over the simulation (minimum radius around 2.5–3 Å), with one observed event of marked widening and successive narrowing. We also generate and refine via MD a model of a double-pore made of eight Cldn15 monomers, which allows us to observe inter-monomer surfaces not present in the single pore and that are relevant for TJ strand formation, specifically the linear *cis* arrangements described above and the interfaces formed by ECL2 segments of different protomers.

Our results demonstrate that the missing protein segments in the model of Suzuki et al. can arrange without clashes in multiple different conformations, and confirm that the structure is a possible arrangement of claudin monomers forming paracellular pores. We thus provide a refined and improved version of the model, in full atomic detail and without missing segments, that can be used in future studies on the molecular mechanism of TJ function.

## Materials and methods

### Preparation of Cldn15 monomer structures

The available crystal structure of Cldn15 (PDB ID: 4P79 [20]) requires some additions and modifications in order to be used in molecular simulations. Specifically, both the monomer crystal structure and the assembled protomers in the TJ model of Suzuki et al. miss few extracellular residues: the crystal lacks eight residues (34 to 41) in ECL1, while the Cldn15 protomer in the TJ model is missing nine residues (34–42) in ECL1 and two (149 and 150) in ECL2. Our strategy comprises the preparation of two distinct Cldn15 protomers for MD simulation.

The first one, named Model1, is a fair replica of the crystallographic structure with *in silico* addition of the missing residues (34–41), modeled by the highest ranked conformation from the loop-closure modeling RCD+ software [29], which uses an *ab initio* algorithm. The full protein structure was further refined with Chimera tools [30], after changing selenomethionines (MSE) to methionines (MET), and using the Dunbrack rotamer library to design missing atoms [31]. Model1 is suitable for MD simulations of the single protomer of Cldn15 and for the assembly of the single-pore system which takes into account only the second *cis* (face-to-face) interface between protomers.

**Fig 3:**
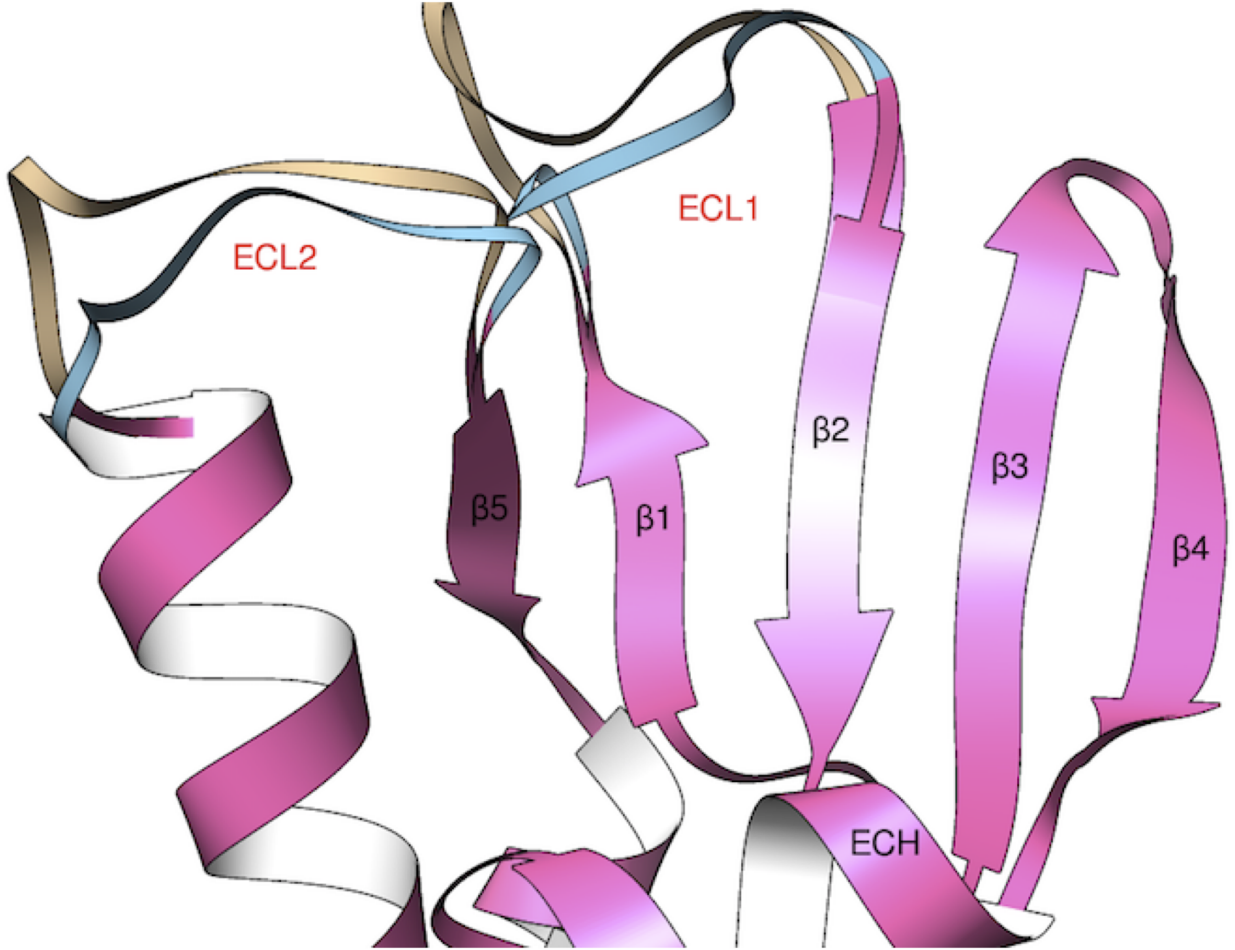
Modeling of Cldn15 loops. Superposition of initial structures of Cldn15 used in the monomer and the single-pore (Model1) and the double-pore (Model2) simulations. The identical folding is pictured in pink, while the two different conformations of loops ECL1 and ECL2 are colored in brown for Model1 and cyan for Model2. Secondary structure elements are labeled in black and red.

For the double-pore simulation, an alternative configuration of the loops of Cldn15 is required. Indeed, as anticipated above, the complete architecture of the model shows head-to-head protomers packing in the regions of missing ECL1 and ECL2 residues and, when these amino acids are directly inserted, steric clashes are created. To overcome this problem, we prepared an alternative structure of Cldn15 (named Model2), where using again RCD+ we generated new coordinates for the residues of ECL1 (34–41) and an extended segment in ECL2 (residues 148–154). We tested different conformations of the two reconstructed regions until we obtained a conformation with non-overlapping extracellular protein regions, suitable to fit the global TJ structure of Suzuki et al. The structure was again further refined using Chimera. The difference between the two final protomer models is highlighted in Fig 3. It is important to stress that the different arrangements of ECL1 and ECL2 in the two models did not alter the crystallographic orientation of the side chains in the linear *cis* interface, since residues F146, F147 and L158 are not part of the reconstructed regions.

### MD simulation of the Cldn15 monomer

In order to benchmark the structure of the Cldn15 monomer and build a reference for protomer analysis in the TJ pore simulations discussed below, we performed all-atom MD simulations of the single protein in an explicit water and lipid environment. The structure described above as Model1 was oriented using the PPM Server [32] in a hexagonal 1-palmitoyl-2-oleoyl-sn-glycero-3-phosphocholine (POPC) bilayer (253 molecules) and solvated with three point (TIP3P) water molecules (17540) and 0.15 M NaCl solution (54 Na ions, 53 Cl ions) neutralizing the net charge of the protein, for a total of 89462 atoms. The conserved disulphide bridge in ECL1 (C52-C62) was maintained in the structure. The membrane builder application of the CHARMM-GUI server [33, 34] was used for the preparation of all the input files of the simulation. Hexagonal periodic boundary conditions were used to replicate the system and remove box surface effects. After an initial energy minimization, the system was heated to 310 K and then simulated using positional restraints on the protein atoms for 2 ns in the NPT ensemble at 310 K and 1 bar. Successively, a 130 ns unrestrained MD simulation was produced in the NPT ensemble at the same temperature and pressure maintained by a Langevin thermostat and Nosé-Hoover Langevin piston pressure control. The NAMD 2.12 program [35] with the CHARMM36 force field [36] was used. During the trajectory, the height of the simulation box is stationary around 103.0 Å, while its hexagonal base is inscribed in a square of about 99.0 × 99.0 Å^2^. Long range electrostatic interactions were calculated using the Particle Mesh Ewald (PME) algorithm [37]. All covalent bonds involving hydrogen atoms (except those of water molecules) were kept fixed using SHAKE [38], and those in water molecules using SETTLE [39]. A time-step of 2 fs was employed. To ensure maximum accuracy, electrostatic and van der Waals interactions were computed at each simulation step. The MD trajectory was visualized and analyzed to validate the structural stability of the Cldn15 protein using Chimera and VMD and associated plugins [40]. In S4 Fig we show the full system in a snapshot extracted from the simulation.

### MD simulations of the single-pore model

#### System set-up and equilibration procedure

**Fig 4:**
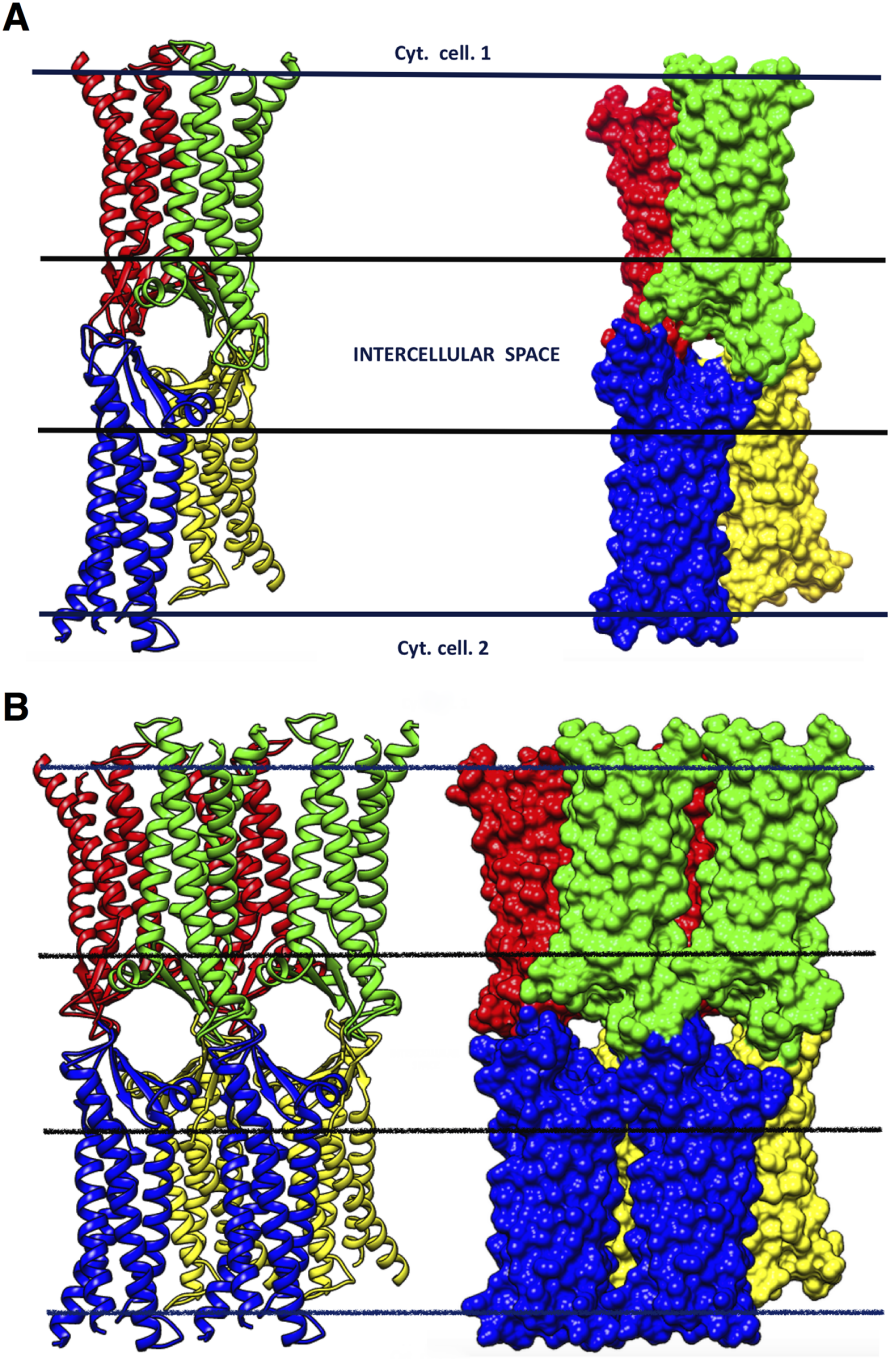
The single and double-pore channel models. **A**, structure of the single-pore system viewed from the apical side, in ribbon (left) and surface representation (right). The complex is formed by the *trans* interaction of two *cis* dimers (red-green, P1-P2 dimer; and blue-yellow, P3-P4 dimer). Horizontal lines indicate the membrane boundaries of two adjoining cells. **B**, Structure of the double-pore system represented and viewed as in **A**. Horizontal lines indicate again the membrane boundaries.

To obtain the starting configuration of the single-pore system, we assembled four replicas of Model1 in the same conformation of the pdb file provided by Suzuki et al. [26] by using the Matchmaker Chimera tool for structure superimposition. Then, the tetramer was energy minimized *in vacuo*, using the GalaxyRefineComplex utility [41], preserving the conformation of the transmembrane domains and relaxing the extracellular domains. The resulting conformation is shown in Fig 4A. We remark that, after the described procedure, the side chains of D55 residues on ECL1 of each protomer still point towards the interior of the channel, consistent with their known regulation of Cldn15 selectivity for cations. Subsequently, the proteins were embedded in a double lipid bilayer to reproduce the TJ paracellular environment. Water molecules at bulk concentration were added in the intercellular space and the two cytosolic regions, and four sodium ions were inserted to neutralize the system charge (one placed in each cytosolic regions and two in the intermembrane space). The configurations of the two bilayers and of the solvent were produced with the CHARMM-GUI server and suitably assembled with the tetramer via Chimera tools, eliminating unfavorable protein-lipid and protein-water contacts. We carefully followed the advices of [42] to optimally combine CHARMM and NAMD input files, for example concerning the orientation of the periodic box.

The final configuration includes four Cldn15 structures (two replicas of the monomer in face-to-face *cis* interaction per side of the junction), 560 POPC molecules (281 in one bilayer and 279 in the other), 26146 water molecules and four sodium ions, for a total of 164814 atoms. VMD tools were used to prepare the topology of the whole system with the CHARMM36 force field, always including the disulphide bridge of ECL1, for each protomer. Hexagonal periodic boundary conditions were used to replicate the system and remove box surface effects. S5 and S6 Figs show the dimensions of the simulation box and the effect of boundary conditions. All other simulation settings were the same of those used for the monomer.

**Fig 5:**
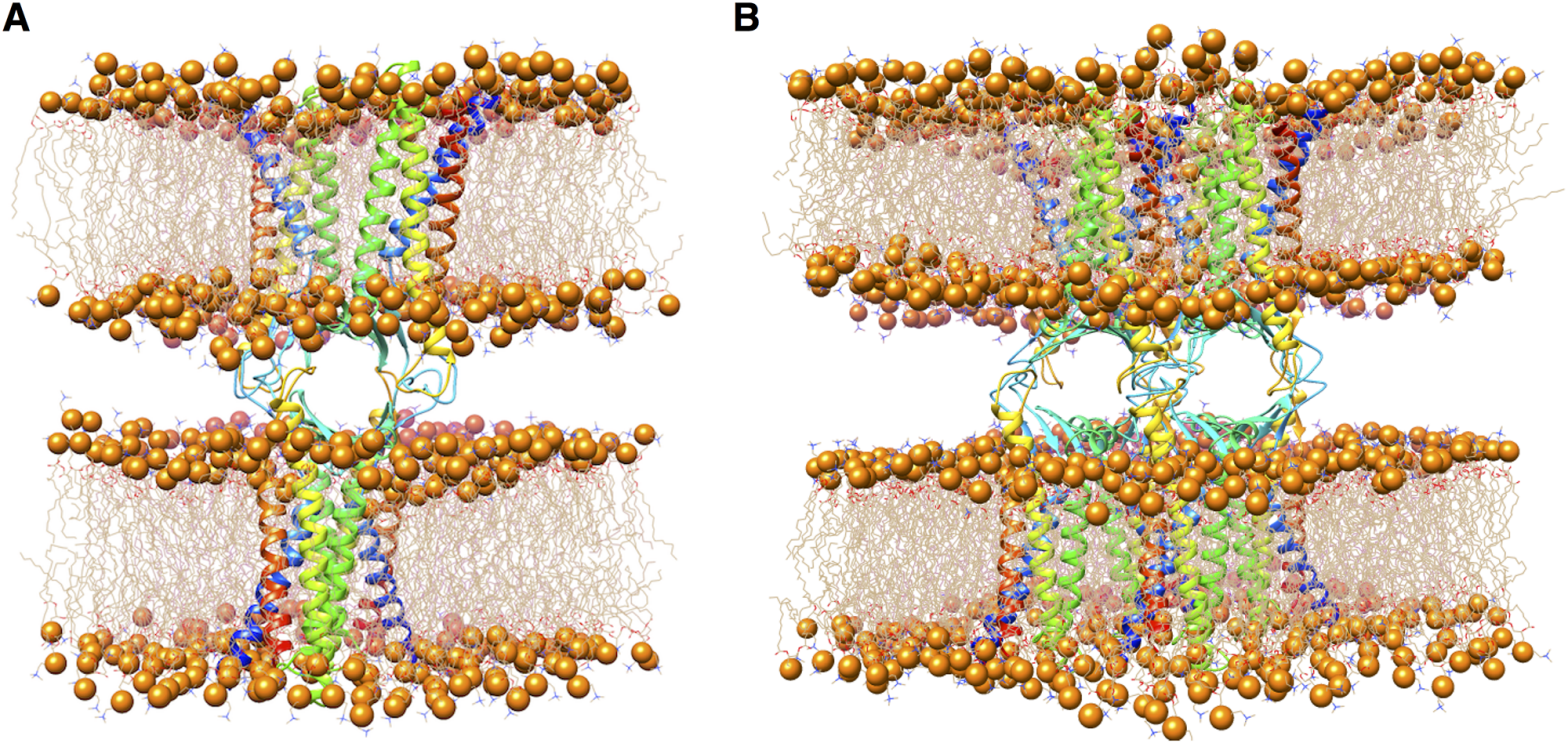
Conformations of single and double-pore systems. The structure of single (**A**) and double (**B**) Cldn15-based paracellular pores, after the respective equilibration protocols. Protomers are shown as ribbons. Each *cis* dimer is embedded in a hexagonal POPC bilayer, shown as wire structures with phosphorus atoms as spheres. Solvent molecules are not shown for clarity.

After an initial energy minimization, the system was heated to 310 K and then simulated for 2 ns in the NPT ensemble (P = 1 bar) with all protein atoms fixed to equilibrate the membrane and water atoms. The system was further equilibrated for 1 ns with the C_*α*_ atoms restrained, and finally for 1 ns with only the *α* helices C_*α*_ atoms restrained. The equilibrated cell box is an hexagonal prism about 162.0 Å high, whose base is inscribed in a square of about 106.0 × 106.0 Å^2^, which ensures a distance larger than 20 Å between adjacent images of the *cis* dimers. In Fig 5A we show a conformation of the single-pore system after the equilibration procedure.

#### Production run

After minimization and restrained equilibration, all restraints were removed and a free MD simulation of the system was produced for 260 ns in the NPT ensemble (T = 310 K, P = 1 bar). The final 250 ns were used for analysis. The MD trajectory was visualized and analyzed to validate the structural stability of the system using Chimera and VMD.

#### Control run

For the same single-pore system, we also performed a replica simulation to monitor the effect of a different equilibration stage. In this replica, after energy minimization and heating to 310 K, a longer equilibration phase was produced: 10 ns with all proteins fixed, 10 ns with only C_*α*_ atoms restrained, and 1 ns with only the C_*α*_ atoms of the transmembrane *α*-helices restrained, always in NPT at 1 bar. Then, the system was simulated in the same ensemble with all restraints removed for 35 ns. No differences were found in the structure obtained with respect to that from the protocol described above for the main simulation. As an illustration, S7 Fig shows the final configuration obtained with this run in comparison with the structure taken from the main production run at the same time frame (35 ns).

### MD simulation of the double-pore model

The structure of Model2 was used for the unrestrained MD simulations of the octameric double-pore model, which includes both the linear and face-to-face *cis* interfaces introduced by Suzuki et al. [26] and described above. Eight replicas of Model2 were superimposed to the pdb file provided by Suzuki et al. [26] by using the Matchmaker Chimera tool. The octamer was energy minimized *in vacuo* to relax the extracellular domains, using the GalaxyRefineComplex utility [41], and restraining the conformation of the transmembrane domains. The resulting conformation is shown in Fig 4B. The octamer was then embedded in a double hexagonal bilayer and solvent was added. The coordinates of the membranes and of the solvent were produced with the CHARMM-GUI server and suitably assembled to the protein ensemble with Chimera tools. Globally, the system includes 8 replicas of Model2 (four replica for face of the junction), 910 POPC molecules (458 for the first membrane and 452 for the second), 63046 water molecules, and 8 sodium ions to neutralize the net charge of the system, for a total of 333750 atoms. An extended equilibration was performed to relax the large structure. After energy minimization and heating to 310 K, the system was submitted to 30 ns total of equilibration in NPT (P = 1 bar) with positional restraints on protein atoms: 6 ns with restraints on the whole proteins, 6 ns with restraints on backbone atoms only, 12 ns with restraints on the C*α* atoms, and finally 6 ns for the C*α* atoms of the *α* helices only, to allow relaxation of the paracellular region.

An unrestrained MD simulation of 35 ns was then produced in the NPT ensemble at 310 K and 1 bar. Along the trajectory, the height of the hexagonal simulation box is stationary around 192.0 Å, while its base is inscribed in a square whose dimensions are stationary around 139.0 × 139.0 Å^2^. The double-pore system after the equilibration procedure is shown in Fig 5B.

### Evaluation of hydrogen bonds survival ratio

Hydrogen bonds (HBs) across the complex interfaces were searched using the VMD software. An HB was considered formed when the donor-acceptor distance and bonding angle were less than 4.0 Å and 30°, respectively. The survival ratio of HBs was defined as the percentage of time the HB was found present along the trajectory.

### Pore size analysis

To study the size of the paracellular channel, we employed the widely used HOLE program [43], which calculates the radius of a protein pore along a given axis by determining the maximum size for a spherical probe that can fit with the van der Waals radii of the atoms. We used a 5 Å threshold for the pore radius which is sufficient to explore the narrow part of the pore. Representative structures spaced by about 10 ns along the simulated trajectory were selected and analyzed.

## Results

### Structural features of the Cldn15 monomer in membrane

**Fig 6:**
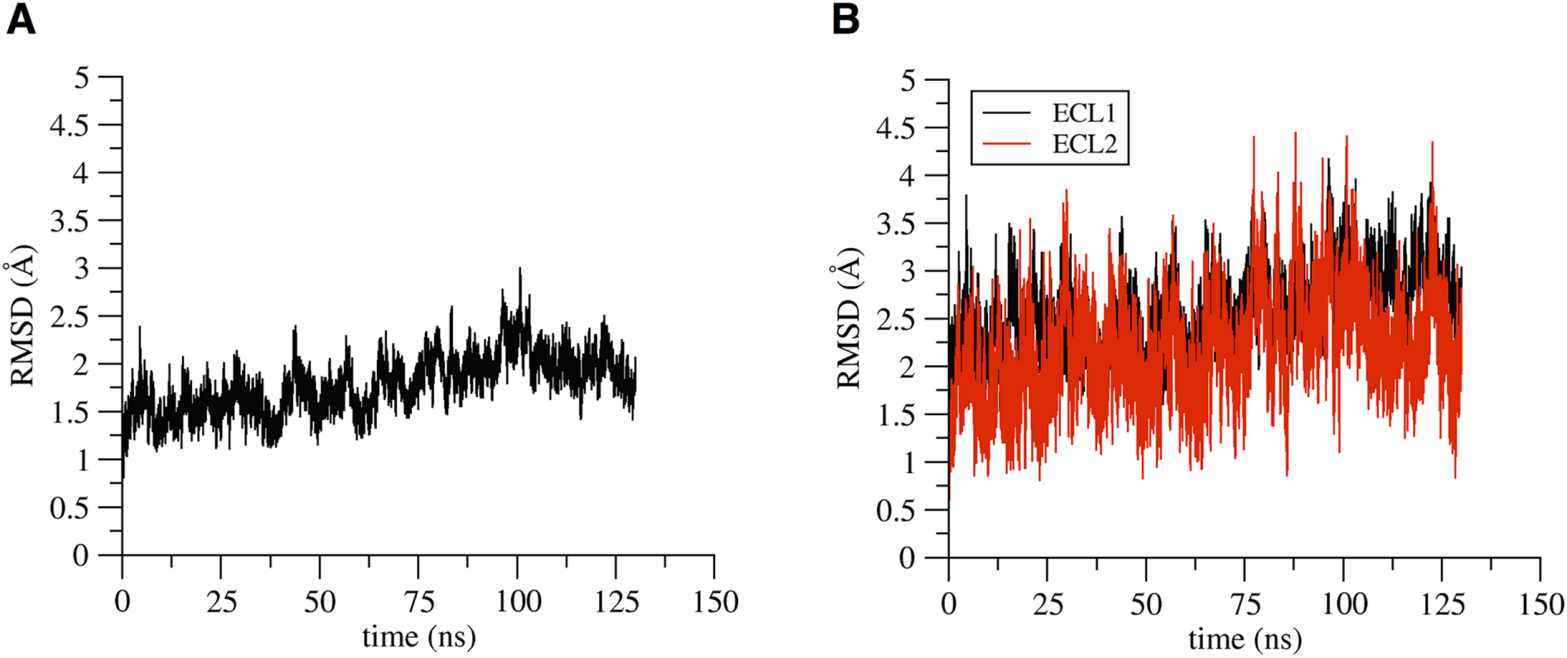
RMSD of Cldn15 monomer. RMSD values of the full protein backbone (**A**), and of the backbone of the two extracellular loops, ECL1 and ECL2 (**B**) along the simulation of the Cldn15 monomer.

We calculated the root-mean-square deviation (RMSD) of atomic backbone positions along the simulated trajectory with respect to the starting conformation, after optimal alignment of the structures. Results show a plateau at about 2 Å for the full protein backbone (Fig 6A) and at about 3 and 2.5 Å for ECL1 and ECL2, respectively (Fig 6B), highlighting the stability of the entire protein structure with limited conformational heterogeneity in the extracellular region. In S8 Fig we report a comparison between the monomer structures in the extracellular region at the beginning and at the end of the trajectory.

The regulation of loop flexibility is based on a pattern of intramolecular HBs which shows some differences with that of the crystal structure described in [20]. A list of HBs survival ratios in the simulated Cldn15 monomer is shown in S1 Table. Some of the bonds present in the crystal structure are not observed during the simulation, such as for example the one between the carbonyl oxygen of P149 and K155, or the one between R79 and F65. Despite the fact that the corresponding residues are in ECL2, the lack of these interactions does not markedly affect the flexibility of the region. On the other hand, R79 on TM2 establishes two persisting HBs with L48 on ECL1 (S9 Fig), also observed in the crystal, which contribute to anchoring the most internal segment of ECL1 to the protein core. It is also relevant to note that there are stable HBs between the two extracellular loops, specifically between *β*5 of ECL2 and *β*1 of ECL1, that maintain the two domains close to each other.

### Analysis of the single-pore model simulation

#### Protein structure and pore conformation

**Fig 7:**
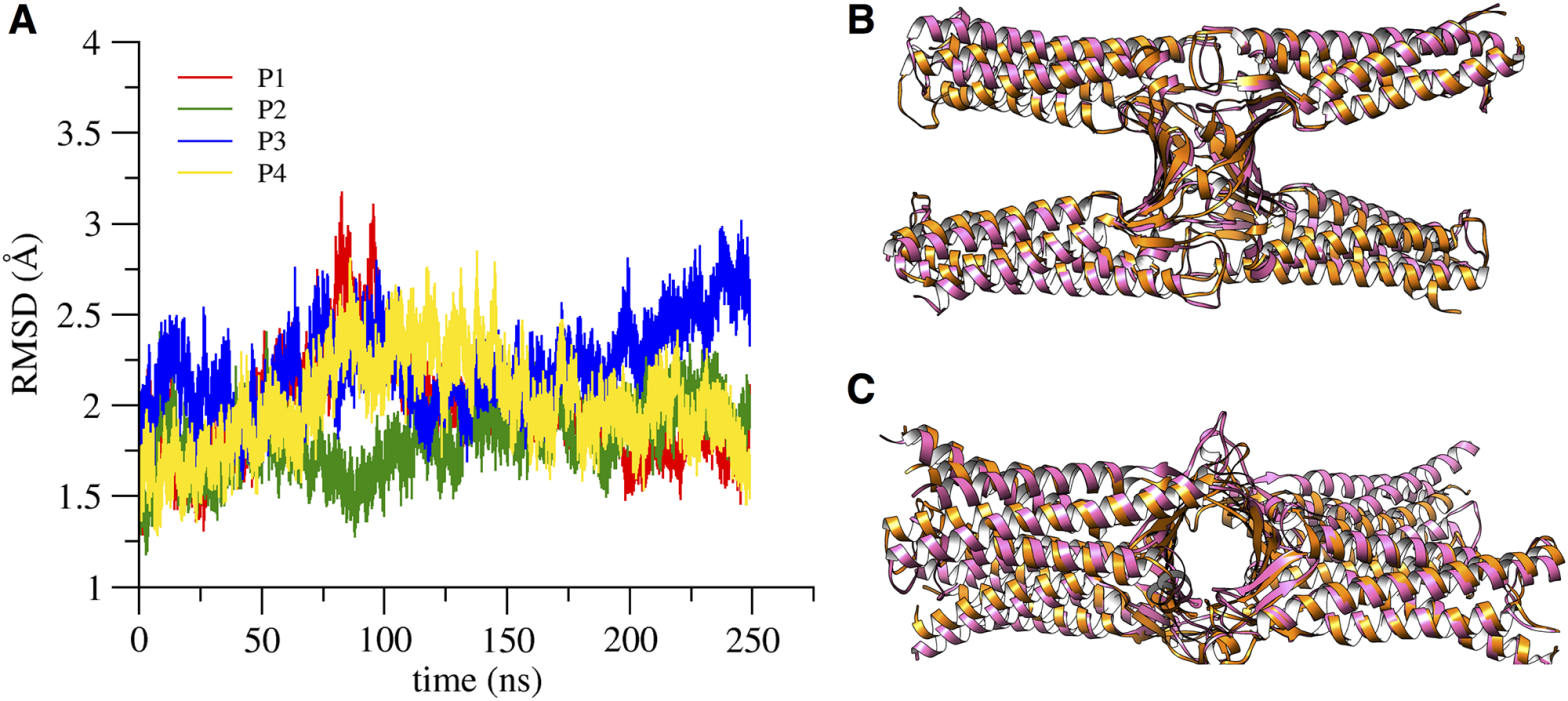
RMSD of the single-pore structure. **A**, RMSD values of the backbone atoms of each protomer in the single-pore simulation. **B** and **C**, superposition of the initial (pink ribbon) and final (orange ribbon) configurations of the channel, viewed from the lateral and apical sides, respectively.

**Fig 8:**
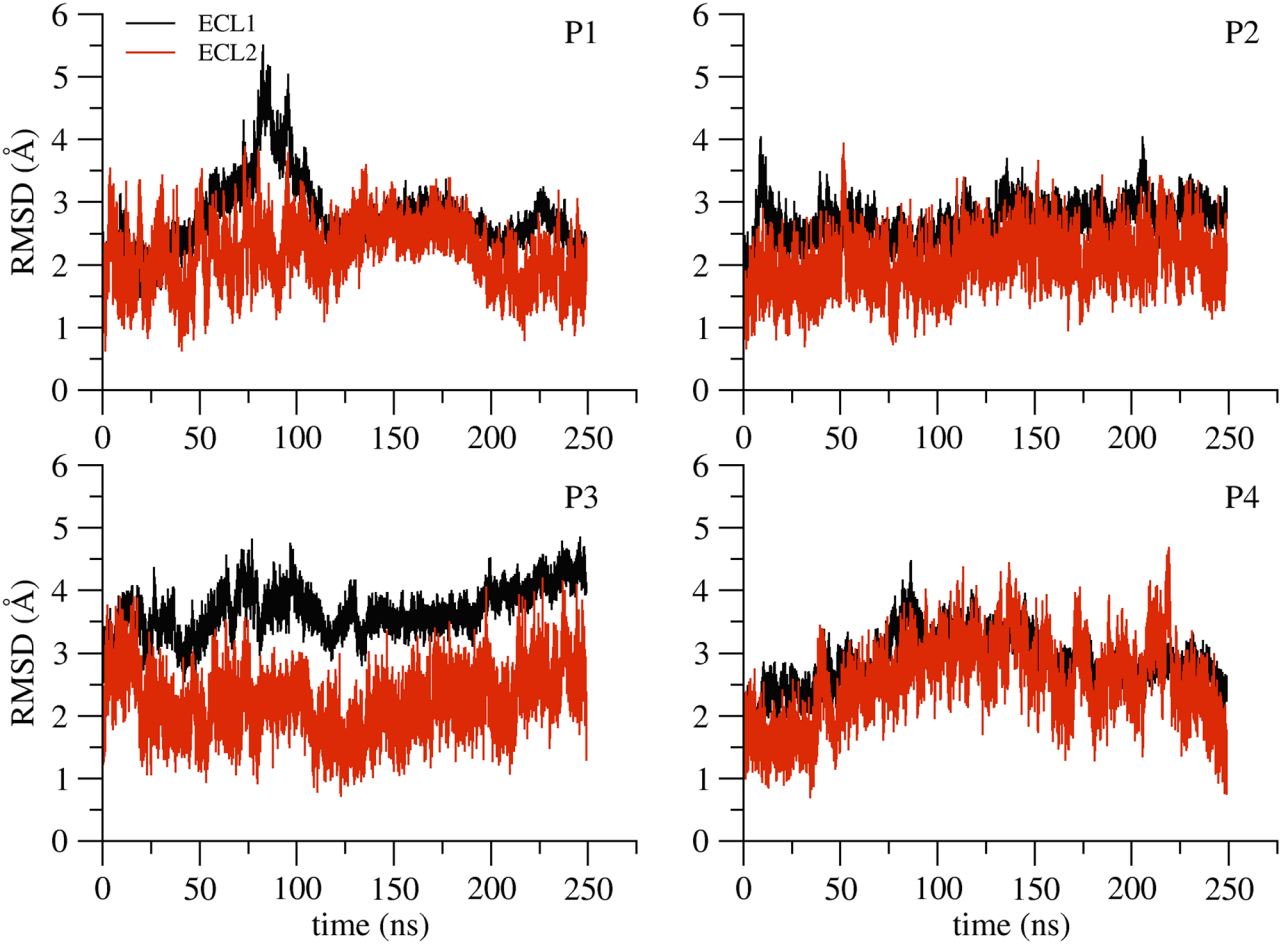
RMSD of the extracellular domains of the single-pore structure. RMSD values of the backbone atoms of the extracellular loops ECL1 (black) and ECL2 (red) in the four protomers along the single-pore simulation.

We calculated the RMSD of backbone atoms along the simulated trajectory with respect to the starting conformation and after optimal structural alignment. The four chains are indicated with names P1 to P4 as reported in S10 Fig. Similarly to what observed for the single protein, RMSD profiles of each protomer show good convergence with plateaus at about 2 Å (Fig 7A), with the exception of P3 where the larger fluctuations are due to movement of ECL1 (see Fig 8 below). During the simulation we observes a slight change in the relative orientation of the protomers, but these limited readjustments do not alter the geometry of the channel, as evidenced below from the analysis of inter-protomer distances and interactions, and can be observed in Fig 7B and 7C, where we compare the structure of the tetramer at the beginning (pink ribbon) and at the end (orange ribbon) of the trajectory.

RMSD profiles of the extracellular loop regions (Fig 8) reveal the flexibility of these domains, which is generally similar to what observed for the monomer. One of the four protomers (P1), however, shows an increase of RMSD values for ECL1 between 75 and 100 ns, and a similar, although less marked, peak can also be observed for P4. However, these deformations appear to be reversible and both protomers return rapidly to a conformation similar to the initial one. The ECL1 of P3 adopts a conformation that is slightly different from the other ECLs, with its tip pointing towards the solvent, but this does not affect the global structure of the pore, as evidenced below.

**Fig 9:**
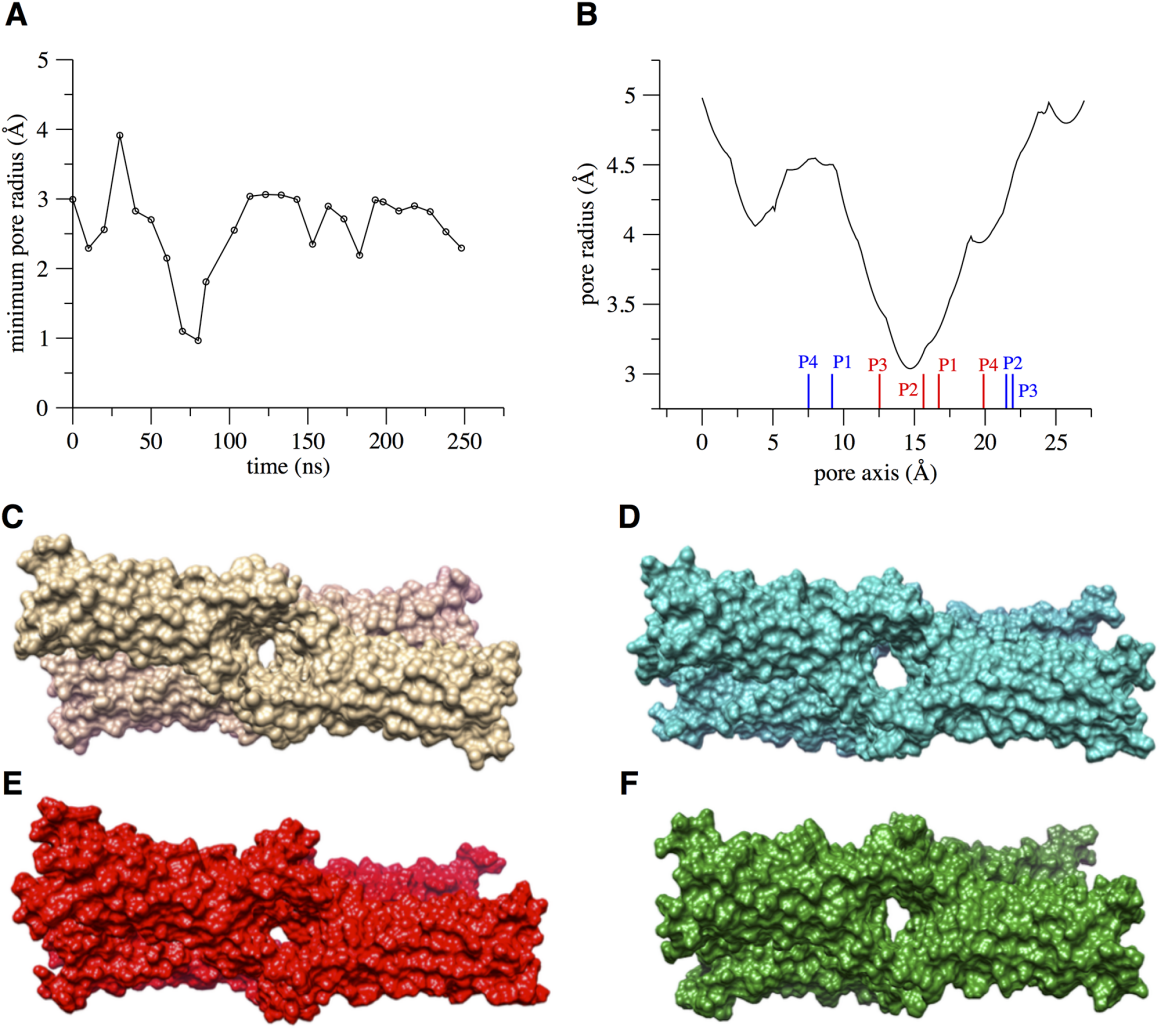
The pore size and structure in the single-pore system. **A**, time evolution of the minimal pore radius along the single-pore simulation. **B**, the pore profile along the channel axis for the configuration taken at 130 ns, with red and blue bars indicating the C*α* positions of D55 and D64 residues, respectively. **C**, the starting conformation of the single-pore model and three snapshots extracted from the simulated trajectory at 30 ns (**D**), 80 ns (**E**), and 130 ns (**F**).

We then analyzed in detail the channel pore features, focusing on the region in the middle of the *β*-barrel structure, indicated as surface in S11 Fig. The minimal value of the pore radius calculated using snapshots of the tetramer taken from the trajectory at approximately each 10 ns, and a representative pore profile calculated at 130 ns (i.e. in the portion of the trajectory that shows less fluctuations of the minimal radius) are shown in Fig 9A and 9B. In Fig 9B, the positions of C*α* atoms of selectivity-determining residues D55 (red bars) and D64 (blue bars) in the four protomers are indicated. It can be seen that the maximal constriction of the pore occurs in correspondence of the D55 residues. The minimal radius is 2.0 Å in the assembled structure before equilibration, and it oscillates around 2.5 Å during the dynamics. Within the first 100 ns, a large fluctuation is observed between between 4 and 1 Å, revealing a breathing motion of the pore. To represent these pore fluctuations visually, in Fig 9 we compare the initial tetramer conformation (**C**) with three structures taken from the simulated trajectory at 30 ns (**D**), 80 ns (**E**) and 130 ns (**F**).

Despite the described fluctuations in pore width, the channel scaffold is quite stable, as can be deduced from visual inspection and from the time evolution of the cross-distances between the C*α* atoms of the four pore-lining C52 residues, located in the middle of the *β*3 strands, which are stationary around 21 Å and show limited fluctuations (see S12 Fig, where the protomers are indicated as in S10 Fig).

#### Interaction analysis of the *cis* and *trans* inter-monomer surfaces

Along the 250 ns MD simulation analyzed, each *cis* dimer retains the face-to-face interaction between *β*4 strands, which are at the basis of the model by Suzuki et al. The presence of stable HBs at this interface is essential to preserve the global stability of the structural model and, in particular to maintain the negatively charged surface of the strand (specifically the side chains of D55 residues) towards the interior of the channel, consistent with the selectivity of Cldn15 for cations.

In Table 1 we report a list of the most persistent *cis* HBs found during the simulation, using the protein chain names introduced in S10 Fig. Stable HBs are formed by *β*4 residues of the two dimers such as S60, N61 and C62. In [26], an enhanced face-to-face dimerization was observed in cross-linking experiments by mutating N61 to a cysteine. Remarkably, the network of HBs is essentially symmetric in the two *cis* interfaces. In particular, two HBs formed between the backbone nitrogen of C62 and the backbone oxygen of C62 of the associate *cis* protomer are the most stable in both dimers. We stress the interesting role of the conserved C62 residue, which contributes to stabilizing each protomer extracellular region via a disulfide bridge with the other conserved cysteine C52, and also forms a persisting HB with the C62 of the *cis* partner, at the basis of the face-to-face interaction interface.

**Table 1:** Persistence of HBs between dimers in *cis* interaction, calculated as percentage of the simulated trajectory.

Dimer 1			Dimer 2		
%	P1 residue (atom)	P2 residue (atom)	%	P3 residue (atom)	P4 residue (atom)
80	C62 (N)	C62 (O)	86	C62 (O)	C62 (N)
74	C62 (O)	C62 (N)	80	C62 (N)	C62 (O)
68	S60 (O)	D64 (N)	74	D64 (N)	S60 (O)
32	N61 (ND2)	N61 (OD1)	66	S60 (O)	D64 (N)
			38	D64 (OD2)	S60 (OG)
			36	N61 (ND2)	N61 (OD1)
			35	N61 (ND2)	C62 (O)
			34	D64 (OD1)	S60 (OG)
			30	D64 (OD2)	S60 (N)

The single-pore dynamics shows how the two *cis* dimers engage stable *trans* HBs, thanks to interactions between the regions of each protomer indicated as V1 in [26] (see S13 Fig). The existence of these favorable interactions had only been hypothesized when the model was first described [26], since various residues of the extracellular zone were missing. In Table 2 we report a list of the most persistent *trans* HBs found along the trajectory.

**Table 2:** Persistence of HBs between dimers in *trans* interaction, calculated as percentage of the simulated trajectory.

P1-P3			P2-P4		
%	P1 residue (atom)	P3 residue (atom)	%	P2 residue (atom)	P4 residue (atom)
50	T41 (OG1)	D55 (OD2)	65	N42 (OD1)	N42 (ND2)
44	T41 (N)	D55 (OD2)	62	N42 (ND2)	N42 (OD1)
			33	T41 (OG1)	T41 (OG1)

The *trans* interface also comprises contacts between hydrophobic residues that seal together facing protomers. In particular, each opposing pair (P1-P4 and P2-P3) establishes ECL2-ECH interactions by bringing the conserved hydrophobic residue A152 (Pro in human Cldn15) close to the facing cluster of conserved residues M68, L69, A70, L71 (S14 Fig), an arrangement that persists for most of the simulation: the distance between the C*α* of A152 and the backbone center of mass of ECH residues 68 to 71 in the facing protomer is stationary around about 7 Å during the whole trajectory for all pairs except P4-P1, where it is so for 75% of the trajectory.

Stable interactions are also formed by hydrophobic residues in the ECL1 segments of diagonally opposed protomers (pairs P1-P3 and P2-P4, S15 Fig). Specifically, L57 in the loop connecting *β*3 and *β*4 is very frequently at a distance smaller than 4 Å from a group of residues in the facing ECL1 which includes the conserved V38, I39 and I44, with percentages of the simulated trajectory that vary among protomer pairs and specific residues, but always fall between 50% and 95%, except for the P4-P2 pair, where L57 is in contact with A152 of ECL2 for 57% of the trajectory.

### Analysis of the double-pore model simulation

#### Protein structure and pores conformation

**Fig 10:**
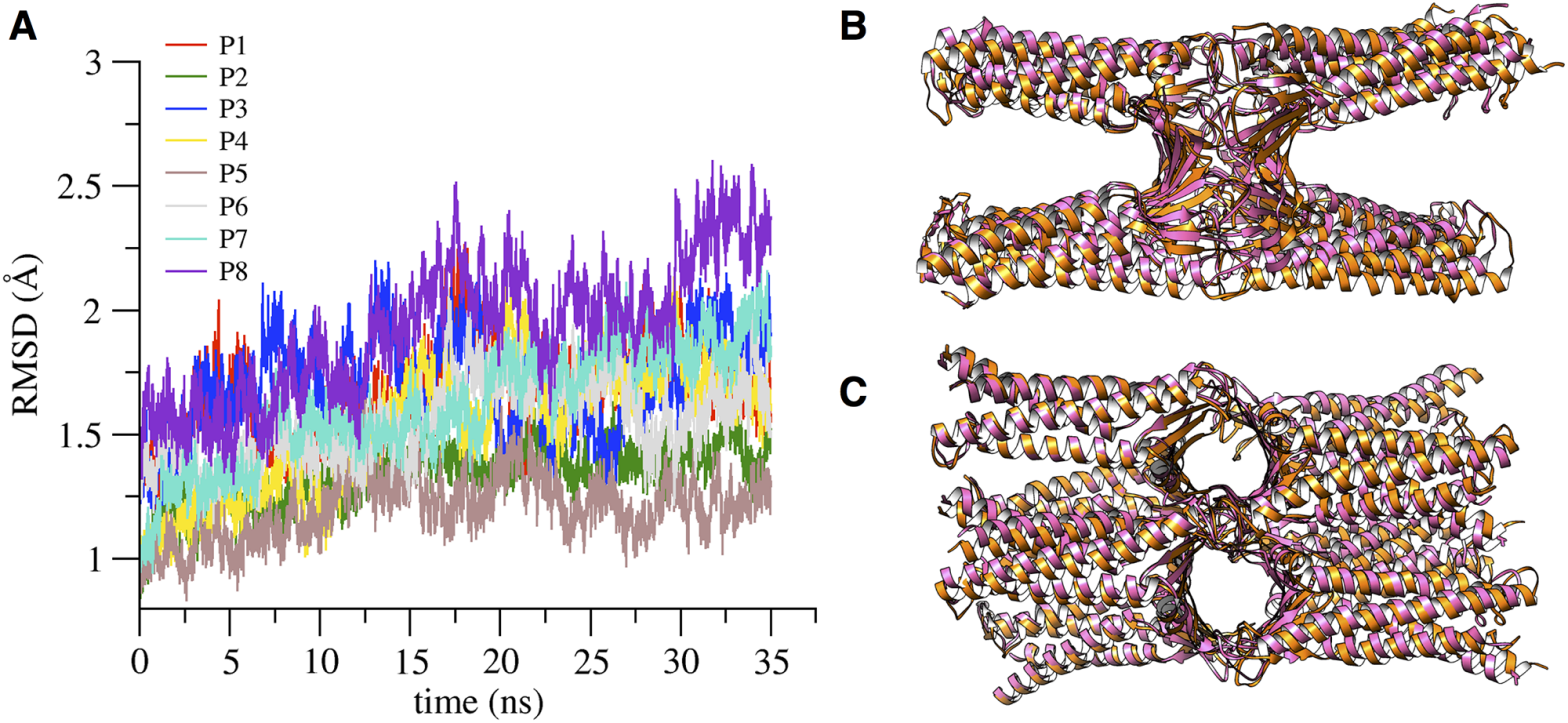
RMSD of the double-pore structure. **A**, RMSD values of the backbone atoms of each protomer along the double-pore simulation. **B** and **C**, superposition of the initial (pink ribbon) and the configuration at 30 ns, viewed from the lateral and apical side, respectively.

We calculated the backbone atoms RMSD with respect to the starting conformation after optimal alignment of structures (Fig 10A). The different chains are indicated with names P1 to P8 following again S10 Fig. The time evolution of each protomer shows values similar to those in the single-pore simulations (compare with Fig 7A), and correlates well with the RMSD profiles of the corresponding extracellular loops (S16 Fig). The architecture of the double channel is well preserved along the trajectory, as can be observed in Fig 10B and 10C, where we compare the double-pore conformation at the beginning of the simulation (pink ribbon) with a snapshot taken at 30 ns (orange ribbon).

**Fig 11:**
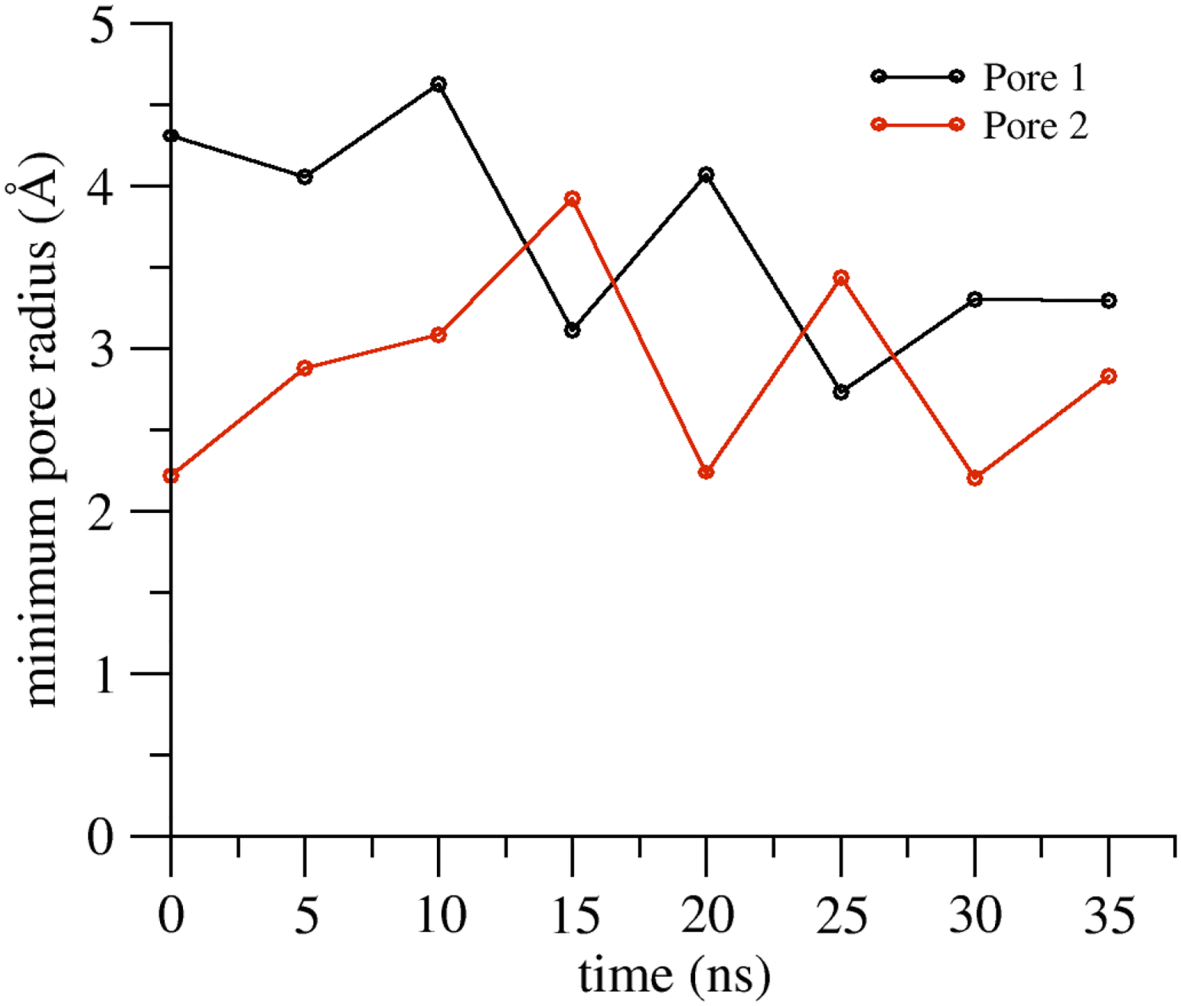
The pore minimal radius of the double-pore structure. Time evolution of the minimal radii of the two pores along the double-pore model simulation.

We analyzed the features of the two channels, focusing again on the region at the middle of the *β*-barrel structure. The values of the minimal radius of both pores along the simulation are shown in Fig 11. The time evolution of the maximal constriction is similar in the two channels and, at least in the short time window observed, it converges to values close to that obtained for the single pore. The scaffold of the double channel is very stable, as suggested by visual inspection (Fig 10B and 10C) and by the time evolution of the cross-distances between the C*α* atoms of the four pore-lining C52 residues in both channels (see S17 Fig).

#### Interaction analysis of the *cis* and *trans* inter-monomer surfaces

We started by analyzing the face-to-face interactions, now occurring between the dimers formed by P1-P2, P3-P4, P5-P6 and P7-P8 (Table 3). Similarly to the tetramer case, each pair shows HBs between residues of the *β*4 barrel such as S60, N61 and C62. The interaction between C62 residues of opposing protomers, already observed in the single-pore simulation, is also detected here for three of the four dimers, while in the missing case (P3-P4) C62 of P3 is in interaction with the C62 preceding residue N61 in P4.

**Table 3:** Persistence of HBs between dimers in *cis* face-to-face interaction, calculated as percentage of the simulated trajectory.

%	P* residue (atom)	P* residue (atom)
74	P6 D64 (N)	P5 S60 (O)
70	P2 C62 (N)	P1 C62 (O)
69	P6 C62 (N)	P5 C62 (O)
67	P5 C62 (N)	P6 C62 (O)
64	P3 C62 (N)	P4 N61 (OD1)
48	P8 C62 (N)	P7 C62 (O)
47	P1 C62 (N)	P2 C62 (O)
40	P2 N61 (ND2)	P1 N61 (OD1)
38	P4 N61 (ND2)	P3 C62 (O)

**Fig 12:**
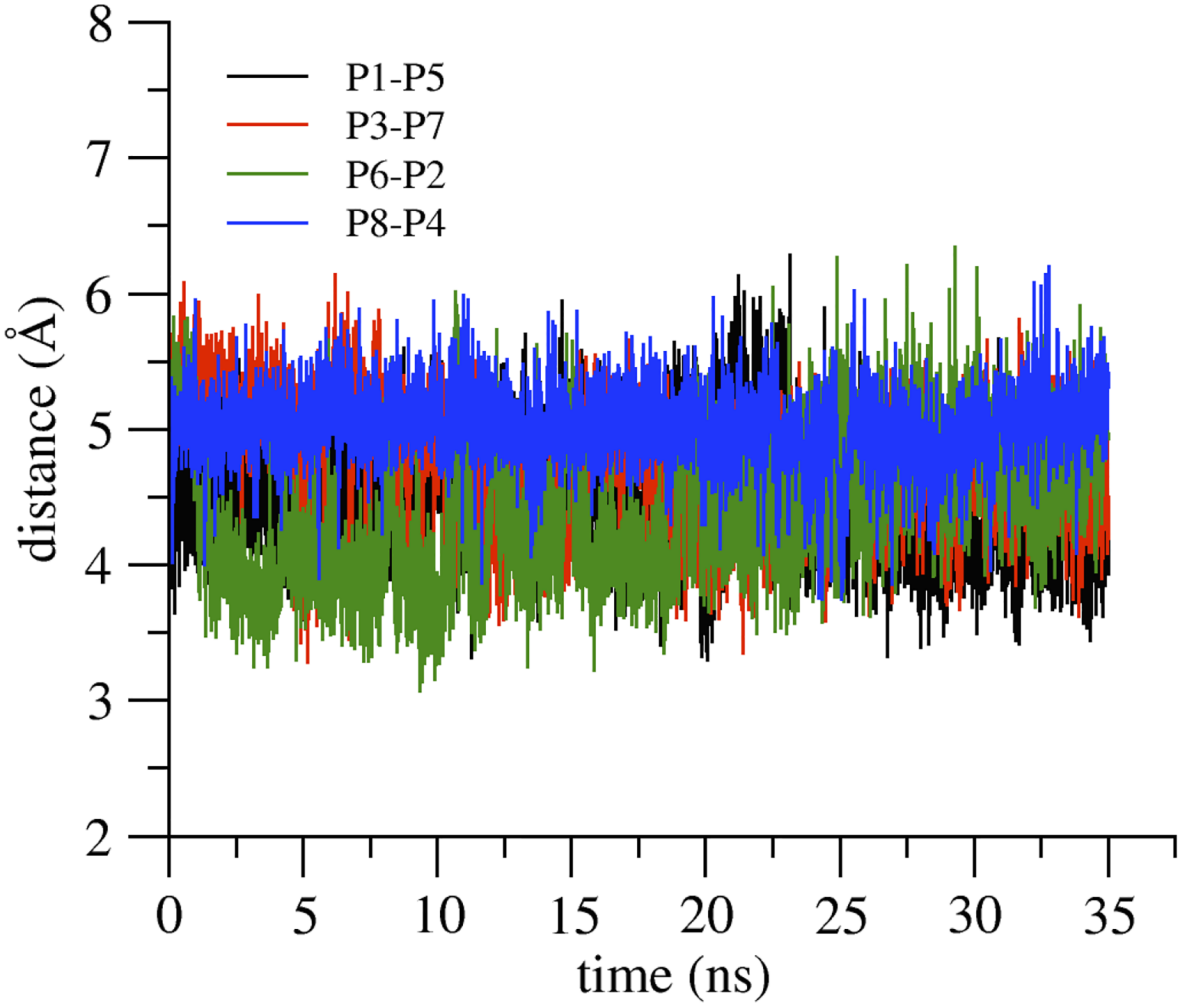
*Cis* linear arrangement of Cldn15 protomers in the double-pore simulation. Distances between the M68 sulfur atom and the center of mass of F146 benzene ring, calculated for the four protomer pairs in *cis* linear interaction. In the legend, the first protomer name in the pair is that of M68.

One relevant feature of the double-pore system is the additional presence of the *cis* linear interacting surface described in the model of Suzuki et al. As already discussed in the Introduction, this motif comprises interactions between the residue M68 of the ECH segment of one protomer with residues F146, F147, and L158 in the TM3 and ECL2 of the adjacent chain. To verify the persistence of this interaction during our double-pore simulation, we calculated the distance between the M68 sulfur atom and the center of mass of the F146 benzene ring for each of the four linear *cis* interacting dimer. Results are shown in Fig 12, and reveal a stable separation along the trajectory, with values that nicely correspond to the main peak of the probability density of Met sulfur-aromatic distances reported by [44] (5 Å), calculated using structures from the Protein Data Bank.

The persistence of HBs for *trans* interactions between protomers is reported in Table 4. Although many involved residues are the same of the *trans* interactions in the single-pore model (compare with Table 2), the specific patterns show differences. This is a consequence of the different modeling we performed for the missing segments in the two systems. However, we remark that, because of the lack of the partecipating residues, these interactions were only hypothesized in the architecture by Suzuki et al., and hence both our patterns fit with the original model structure.

**Table 4:** Persistence of HBs between dimers in *trans* interaction, calculated as percentage of the simulated trajectory.

%	P* residue (atom)	P* residue (atom)
84	P5 T41 (OG1)	P4 T40 (O)
83	P5 T40 (N)	P4 V34 (O)
65	P5 N37 (N)	P4 N37 (O)
60	P4 H35 (ND1)	P5 V38 (O)
53	P5 N37 (ND2)	P4 T40 (OG1)
49	P6 T41 (N)	P8 S56 (O)
46	P7 T40 (OG1)	P5 D55 (OD1)
45	P5 T40 (OG1)	P7 S56 (O)
40	P7 H35 (ND1)	P2 V38 (O)
36	P7 N37 (N)	P2 V34 (O)

**Fig 13:**
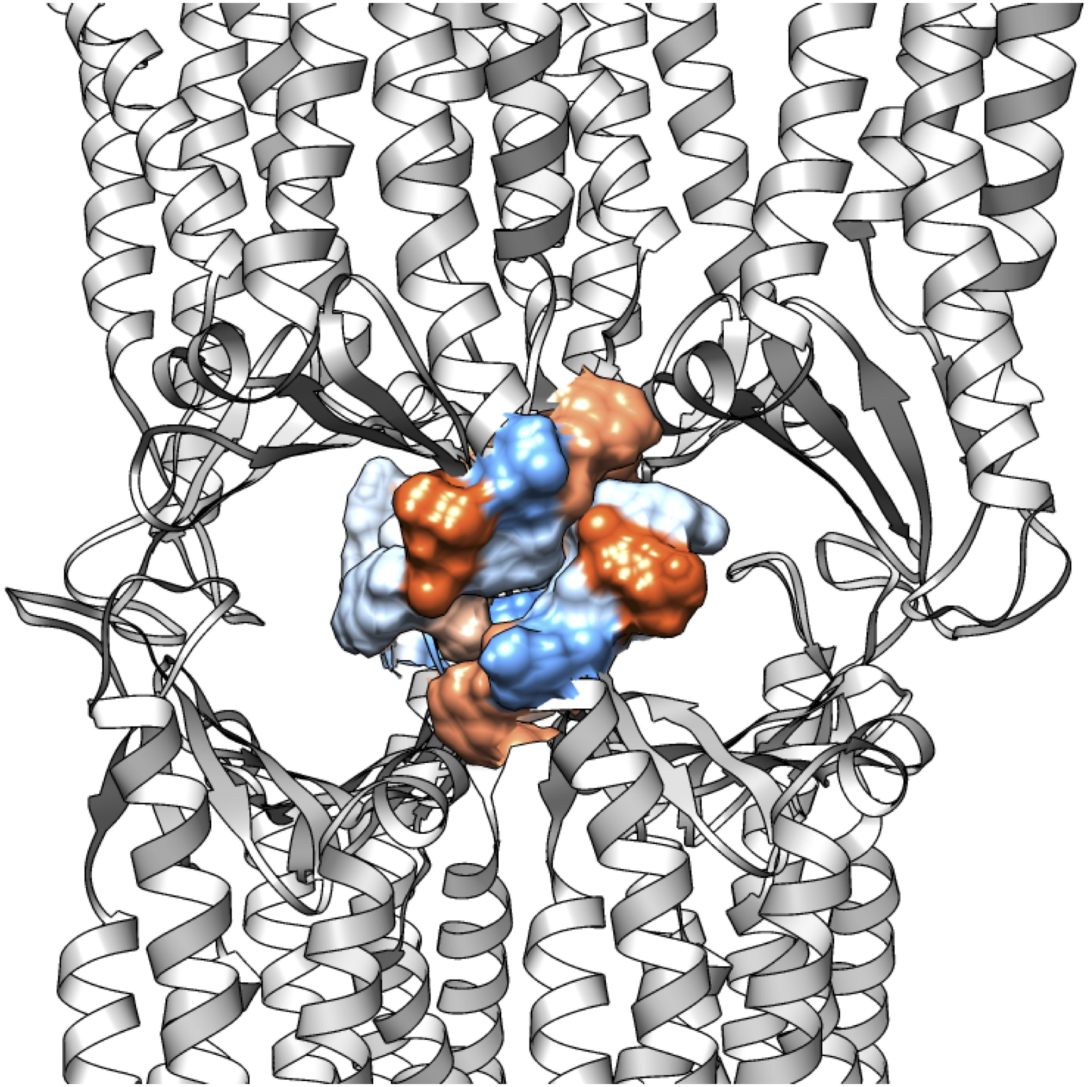
Hydrophobic *trans*- interactions in the double-pore model. Representation of the *trans-* ECL2-ECL2 interactions in the double-pore structure, as observed in a snapshot taken at the end of the simulation. Residues 146 to 155 of each facing protomer are depicted as surface, and colored by degree of hydrophobicity following the Kyte-Doolittle scale as implemented in Chimera: from blue (most hydrophilic, less hydrophobic) to white, to orange (less hydrophilic, most hydrophobic).

Finally, in the double-pore model we can also analyze *trans* interactions between ECL2 segments of facing protomers that were absent in the single-pore. Indeed, in [26] it is hypothesized that the missing region V2, part of ECL2, plays a primary role in the formation of multi-pore strands between claudins from adjacent cells and, in [27], it is stated that ECL2 has an anchoring function for *trans* interactions in TJ strands. In our starting double-pore model we observed a dense packing of hydrophobic residues from symmetrically facing ECL2s, including F146, F147, L150, A152, G153. Remarkably, this cluster is preserved during the simulation, as illustrated in Fig 13.

## Discussion

Understanding the molecular mechanisms at the basis of biological barriers function is a target of pivotal importance in molecular biology and pharmacology. Indeed, crossing the epithelial sheet that forms the barrier is a much sought route for direct drug delivery to pathological sites [10]. TJs are molecular architectures that link adjacent epithelial cells by forming a network of strands on each cell wall, and regulate paracellular diffusion of solutes across barriers. Many proteins form the TJ complex, such as occludins, junctional-adesion molecules (JAM), or Zonula occludens-1 (ZO-1). However, the structural and functional properties of TJs are mainly ascribed to members of the claudin family.

Structural studies of claudins have only recently started to help dissecting the molecular architecture of TJs and the mechanism of their function. At the moment, there are only three available crystal structures of claudins: Cldn15, and Cldn4 and Cldn19 complexed with the same enterotoxin fragment.

Based on the Cldn15 structure, Suzuki et al. [26] have proposed a model of paracellular TJ channels formed by linear polymers of claudins assembled within the membrane into antiparallel double rows, and associating across facing membranes to compose paracellular *β*-barrel-like pores. The detailed atomic model is consistent with crosslinking and mutational experiments and fits with EM images, but misses residues in claudin extracellular loops that, once added, generate steric overlaps. For these reasons, it is so far considered a valuable starting hypothesis that needs further testing [27].

In this work we refined, via structural modeling and all-atom MD simulations, the assembly of Suzuki et al., to provide high resolution, integral chain structures of claudin-based paracellular channels in explicit double membrane bilayer and solvent environment. Starting from the Cldn15 crystal structure and arranging different replicas according to the suggestion of Suzuki et al., we generated single-pore tetrameric and double-pore octameric architectures of claudin protomers. The fragments containing missing residues were modeled and optimized in order to fit with the assembled structure.

In both of our MD refined models, the architecture of the channels was well maintained, as revealed by overall stability of the protomers structure, preservation of the *β*-barrel-like pore scaffold (measured also via distances between backbone atoms of facing residues across the channel), and maintenance of the *cis* and *trans* interacting surfaces between protomers [26, 27]. In the more extended trajectory of the single-pore model, we observed large fluctuations in minimal pore radius within 0–100 ns, followed by stabilization around 2.5 Å up to 250 ns, while the double pore system showed rapid convergence around 3 Å for the minimum radius of both pores. This results in a maximal pore constriction of 5–6 Å, in agreement with electrophysiological studies performed on another pore-forming claudin, Cldn2, where the formation of tight pores of 6.5 Å diameter at the narrowest point was suggested [19].

By analysis of HB lifetime and other interactions patterns we confirmed the relevant role of specific residues in establishing protomer-protomer interacting interfaces. For the *cis* face-to-face interface, the *β*4 strands engage persistent HBs which include S60, N61 and C62 residues. The relevance of this protein region for dimeric interactions is highlighted in [26], where it was observed that N61C Cldn15 mutants are expressed as dimers. The double pore model simulation also allowed observing the linear *cis* interaction surface formed by ECL2 and ECH. This motif was suggested based on the Cldn15 crystal packing [20], and contains ECL2 residues whose corresponding ones in Cldn5 and in Cldn3 have been shown to regulate TJ strand formation [27]. For Cldn15, the role of M68, F146 and F147 has been observed by freeze fracture EM, where mutations to smaller or charged residues at these sites hampered strand formation [20]. In our trajectory, we observed a very stable interaction, as revealed by distances between the M68 sulfur atom and the center of mass of F146 benzene ring that are stationary around 5 Å. Most importantly, our simulations allow to investigate for the first time the formation of *trans* interactions between dimers that involve the missing protein segments in the model by Suzuki et al. This analysis reveals that ECL1 and ECL2 residues engage persistent HBs and hydrophobic interactions with conserved residues of facing protomers.

As a final remark, while the single-pore structure provides a reduction in system size that is advantageous for MD simulations, it is still of general interest since it is known that the main molecular determinants of TJ selectivity are at the individual pore level. Consistently, some studies of TJ electrophysiology are based on single-pore conductance models [19, 45, 46]. Our double-pore system comprises structural features that complement those of the single-pore and that are responsible for TJ strand formation.

## Conclusions

In this work, we have presented atomic detailed structural models of Cldn15-based paracellular TJ pores, obtained via computational modeling and fully explicit MD simulations. Starting from the assembly suggested by Suzuki et al. [26], we have generated a single-pore and a double-pore structure, both embedded in double membrane bilayers. Our results show that Cldn15 protomers can fit the putative architecture of the channel after insertion of the segments that were missing in the original assembly. Hence, we provide an improved and refined version of the model that captures several features described by the available experimental data. *Cis* and *trans* interaction surfaces between protomers are well conserved along the simulation by establishment of hydrophilic and hydrophobic contacts, some of which involve residues whose role was confirmed experimentally. Although pore-size affecting fluctuations are observed in the extracellular loops, the channel architecture is stably maintained. The minimal pore size shows an average diameter of 5–6 Å, nicely fitting experimental data for other claudin pores that tightly control paracellular permeation. In conclusion, our work supports the validity of the original structural model and yields an ameliorated version suitable for studies aimed at dissecting the finest details of TJ function.

## Notes

All figures were prepared with VMD and UCSF Chimera.

## Supporting information

S1 Table Percentage of selected HBs formed between Cldn15 residues. (PDF)

S1 Fig Linear arrangement of Cldn15 protomers in the crystal structure. Representation of the crystallographic linear arrangement of Cldn15 protomers (ribbon representation) aligned along the crystal b axis, viewed from the extracellular space (panel **A**) and from the membrane (panel **B**). The red square in panel **B** highlights the region of the lateral interaction between protomers and panel **C** shows a zoom of the region where the critical residues involved are introduced in stick style.
(PDF)

S2 Fig Double *cis* arrangement of Cldn15 protomers in the Suzuki model. **A**: protomers (green, cyan and purple) aggregate antiparallel with another linear group of claudins (gold) via a second *cis* interface (*face-to-face*) formed by the close vicinity of *β*4 strands and highlighted in **B**.
(PDF)

S3 Fig Extracellular domains of the Cldn15 protomer in the Suzuki model. Disordered loops that might cause steric clashes are not shown (ECL1 residues 34–42 and ECL2 residues 149–150).
(PDF)

S4 Fig Equilibrated configuration of the Cldn15 monomer. Viewed from the transmembrane domain (left) and from the extracellular environment (right). Cldn15 monomer (rainbow cartoon) is embedded in a POPC bilayer, shown as wire structures with sphere phosphorus atoms. Water molecules and ions are not shown for clarity.
(PDF)

S5 Fig Periodic boundary conditions along the *z* axis of the single-pore system. The unit box is indicated by gray lines and periodically repeated along *z*. Shown are POPC lipids in light gray (with phosphate atoms in brown), water (red), and the Cldn15 protomers of the channel in VDW style coloured cyan. Approximate dimensions for each compartment are indicated.
(PDF)

S6 Fig Periodic boundary conditions in the *x* − *y* plane of the single-pore system. The unit box is shown as VDW spheres, while in the replicas lipids are pictured as gray lines and the the protein as ribbon with purple transmembrane domain and yellow extracellular region. Solvent molecules are not reported for clarity.
(PDF)

S7 Fig Single-pore control simulation. Superposition of the final configuration of the control simulation of the single pore structure (orange ribbons) and the structure taken from the main production run at the same time frame, ∼ 35 ns, (pink ribbons). **A** parallel with, and **B** perpendicular to, the elongation of the TJ strand.
(PDF)

S8 Fig Cldn15 monomer simulation. Superposition of the Model1 structure (cyan) and the final configuration from the MD run (brown) of Cldn15 monomer.
(PDF)

S9 Fig Cldn15 monomer hydrogen bonds. The side chain of R79 establishes two HBs with the main-chain carbonyl group of L48.
(PDF)

S10 Fig Chain labeling in single and double-pore systems. Ribbon representation of the single pore (left) and double pore (right) systems, with the labels of protomer segnames, used for the data analysis.
(PDF)

S11 Fig Pore cavity region of the single-pore structure. (PDF)

S12 Fig Cross-distances between facing C52 C*α* atoms in the single-pore system. (PDF)

S13 Fig Relevant HB *trans* interactions in the single-pore simulation. (PDF)

S14 Fig Hydrophobic contacts in the single-pore system. Contacts between the conserved residue A152 of P1 protomer and the conserved residues M68, L69, A70, L71 of the ECH region of P4 protomer.
(PDF)

S15 Fig Hydrophobic contacts of ECL1 segments in the single-pore system. Hydrophobic interactions between ECL1 segments of diagonally opposed protomers. Specifically, L57 of P2 protomer is in close contact with the group of residues V38, I39 and I44 of P4 protomer.
(PDF)

S16 Fig Backbone RMSD of ECL1 and ECL2 in the double-pore simulation. (PDF)

S17 Fig Cross-distances between facing C52 C*α* atoms in the double-pore system. (PDF)
