# Correction: A refined model of claudin-15 tight junction paracellular architecture by molecular dynamics simulations

**DOI:** 10.1371/journal.pone.0193383

**Published:** 2018-02-20

**Authors:** Giulio Alberini, Fabio Benfenati, Luca Maragliano

The following information is missing from the Funding section: This project has received funding from the European Union’s Horizon 2020 research and innovation programme under grant agreement No. 696656 –GrapheneCore1.
