# Supplementary material for: A refined model of claudin-15 tight junction paracellular architecture by molecular dynamics simulations
Source: PLoS One. 2017 Sep 1;12(9):e0184190. doi: 10.1371/journal.pone.0184190 (PMC5581167; doi:10.1371/journal.pone.0184190)
Supplement: S1 Table — (PDF) [file pone.0184190.s001.pdf]

**residue - atom (position)**	**residue - atom (position)**	**%**
R79 - NH2 (TM2)	L48 - O (ECL1)	53
R79 - NE (TM2)	L48 - O (ECL1)	32
V31 - N (ECL1)	E157 - O (ECL2)	90
V31 - O (ECL1)	E157 - N (ECL2)	85
T33 - N (ECL1)	K155 - O (ECL2)	75
W29 - O (ECL1)	G159 - N (ECL2)	43
T33 - OG1 (ECL1)	E157 - OE2 (ECL2)	42
T33 - OG1 (ECL1)	E157 - OE1 (ECL2)	41
