# Supplementary figures and images for: A refined model of claudin-15 tight junction paracellular architecture by molecular dynamics simulations
Source: PLoS One. 2017 Sep 1;12(9):e0184190. doi: 10.1371/journal.pone.0184190 (PMC5581167; doi:10.1371/journal.pone.0184190)

**A**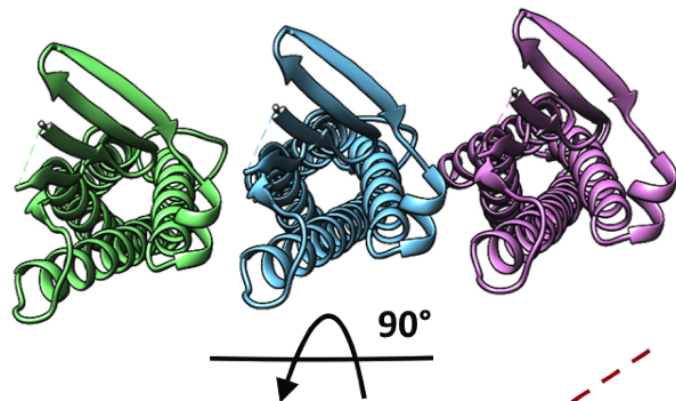**B**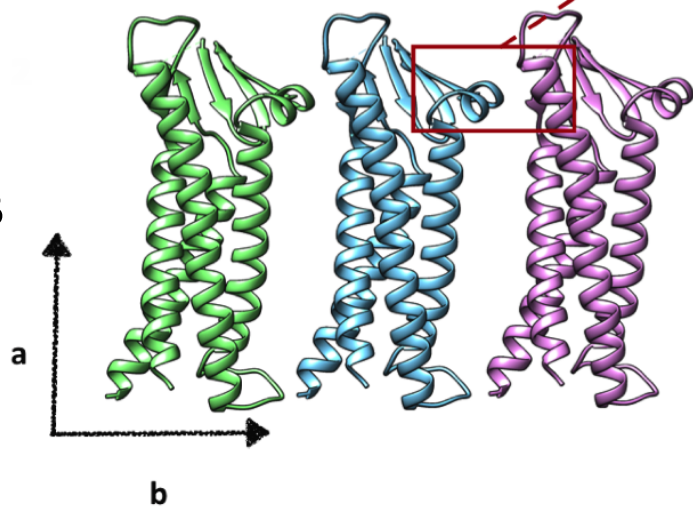**C**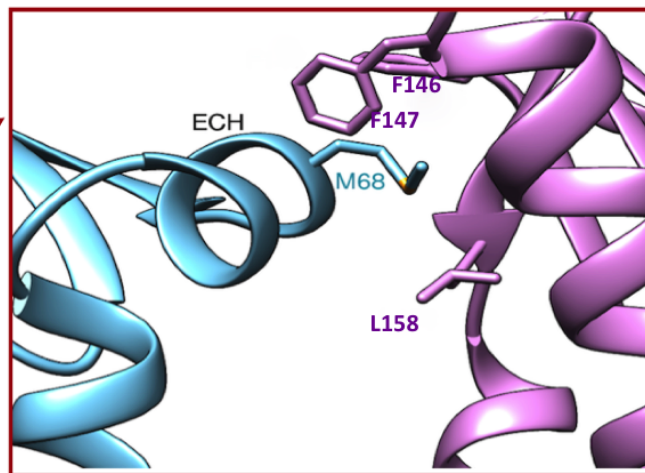

Supplement: S1 Fig — Representation of the crystallographic linear arrangement of Cldn15 protomers (ribbon representation) aligned along the crystal b axis, viewed from the extracellular space (panel A) and from the membrane (panel B). The red square in panel B highlights the region of the lateral interaction between protomers and panel C shows a zoom of the region where the critical residues involved are introduced in stick style. (PDF) [file pone.0184190.s002.pdf]

**A**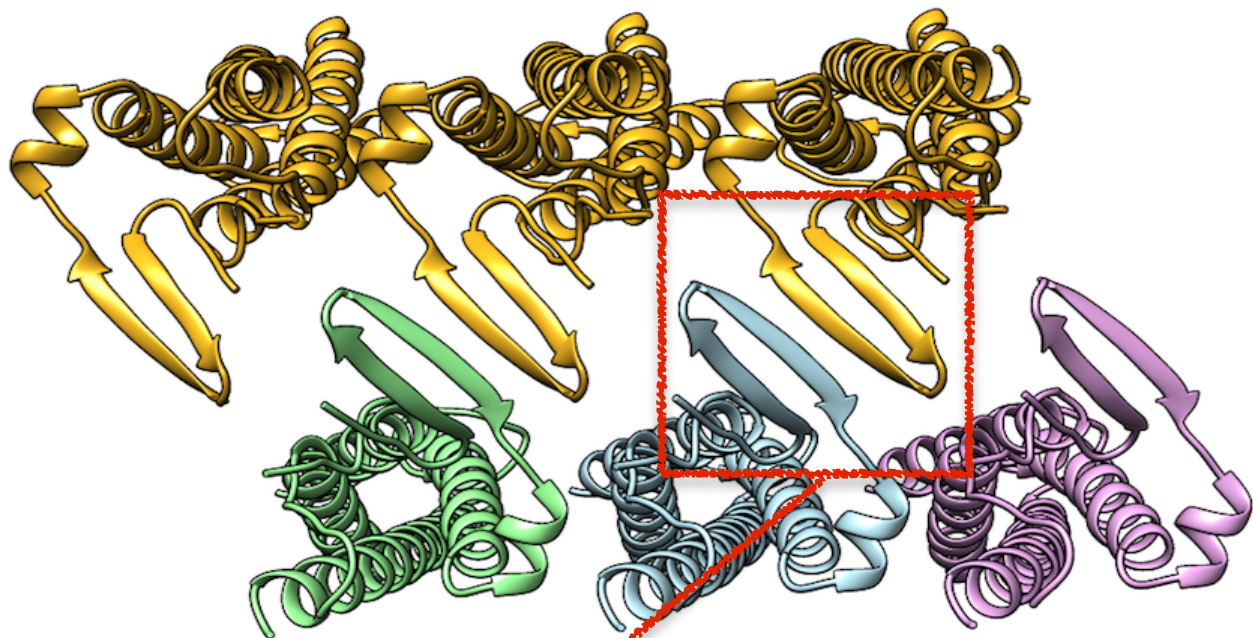**B**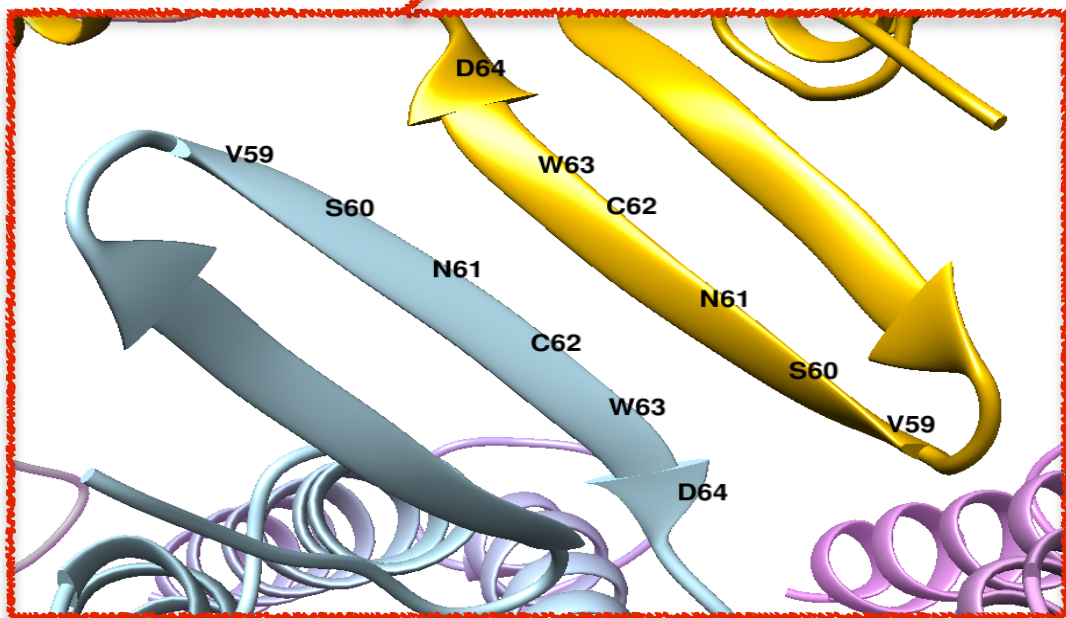

Supplement: S2 Fig — A: protomers (green, cyan and purple) aggregate antiparallel with another linear group of claudins (gold) via a second cis interface (face-to-face) formed by the close vicinity of β4 strands and highlighted in B. (PDF) [file pone.0184190.s003.pdf]

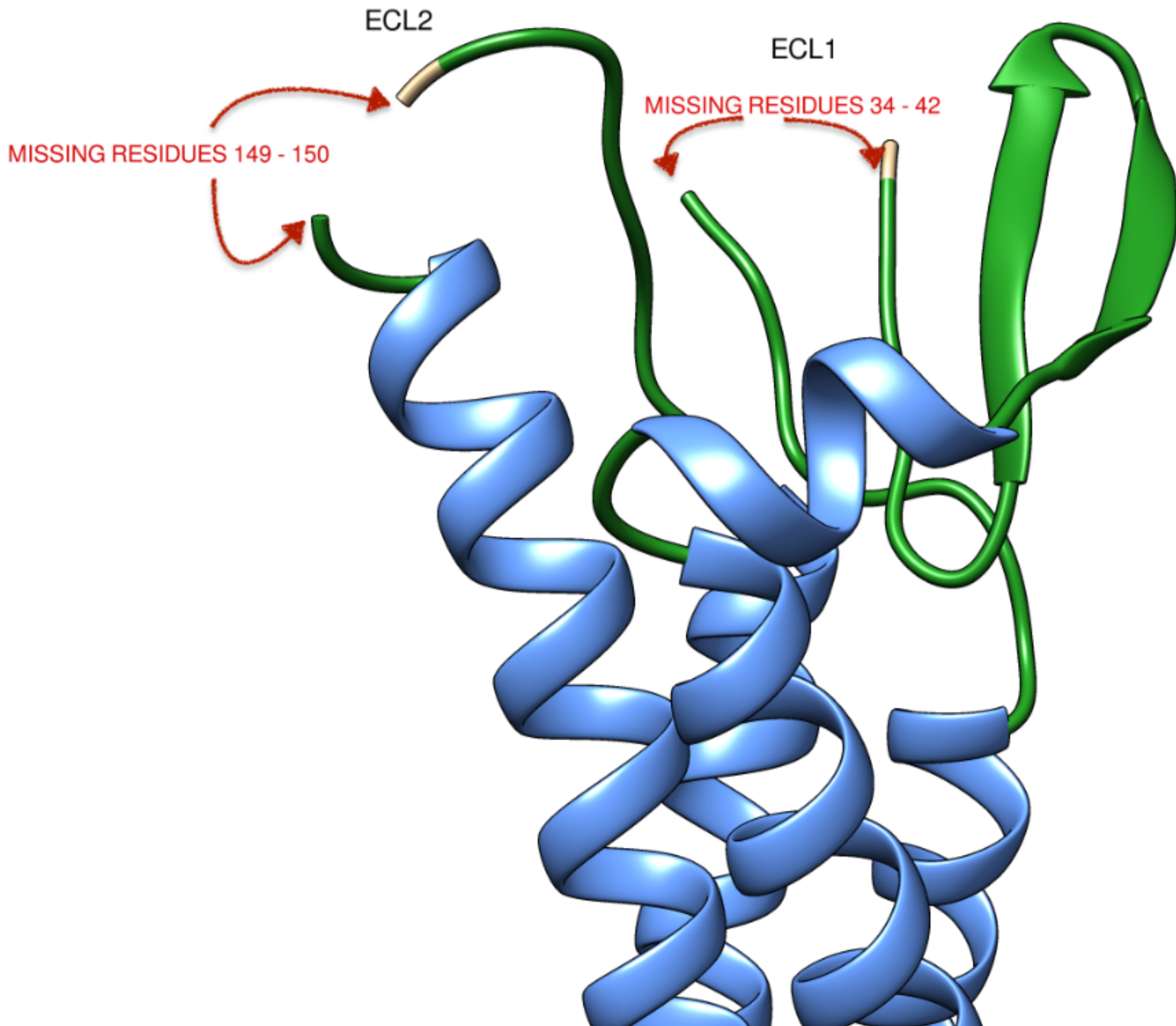

Supplement: S3 Fig — Disordered loops that might cause steric clashes are not shown (ECL1 residues 34–42 and ECL2 residues 149–150). (PDF) [file pone.0184190.s004.pdf]

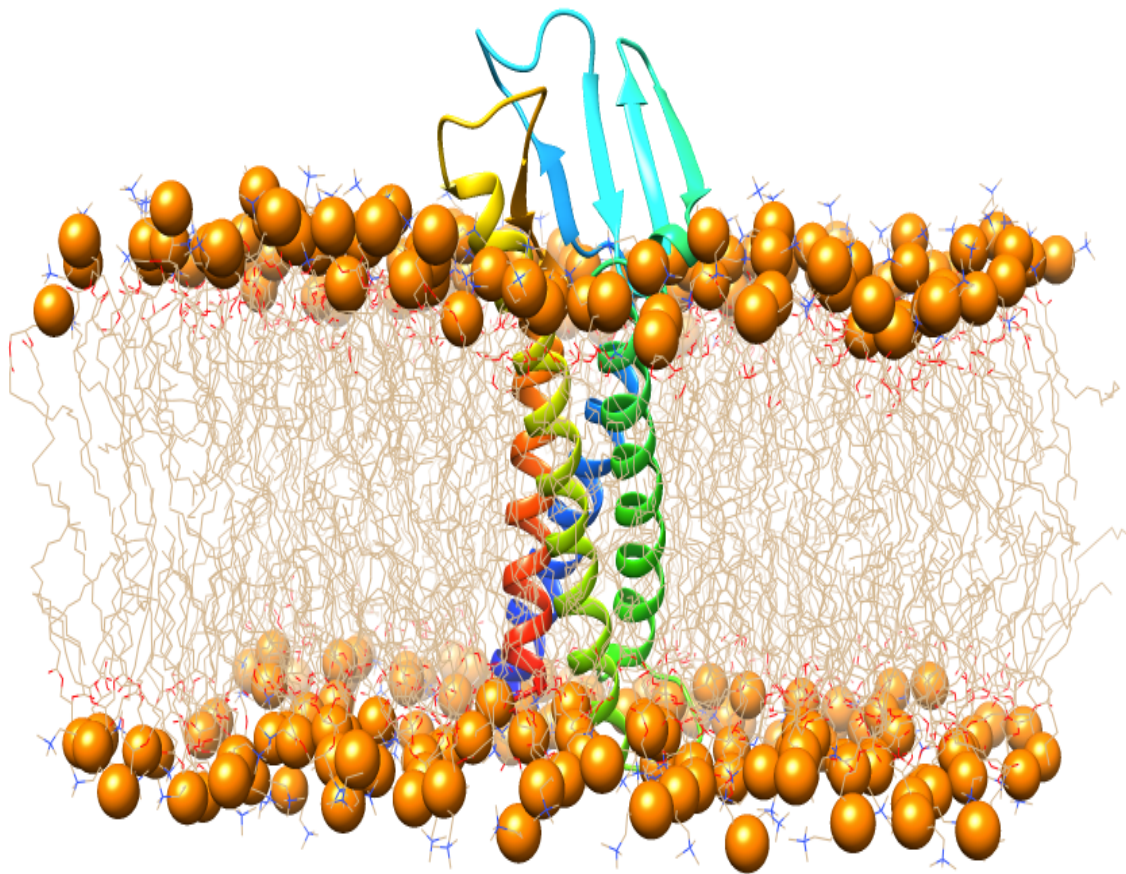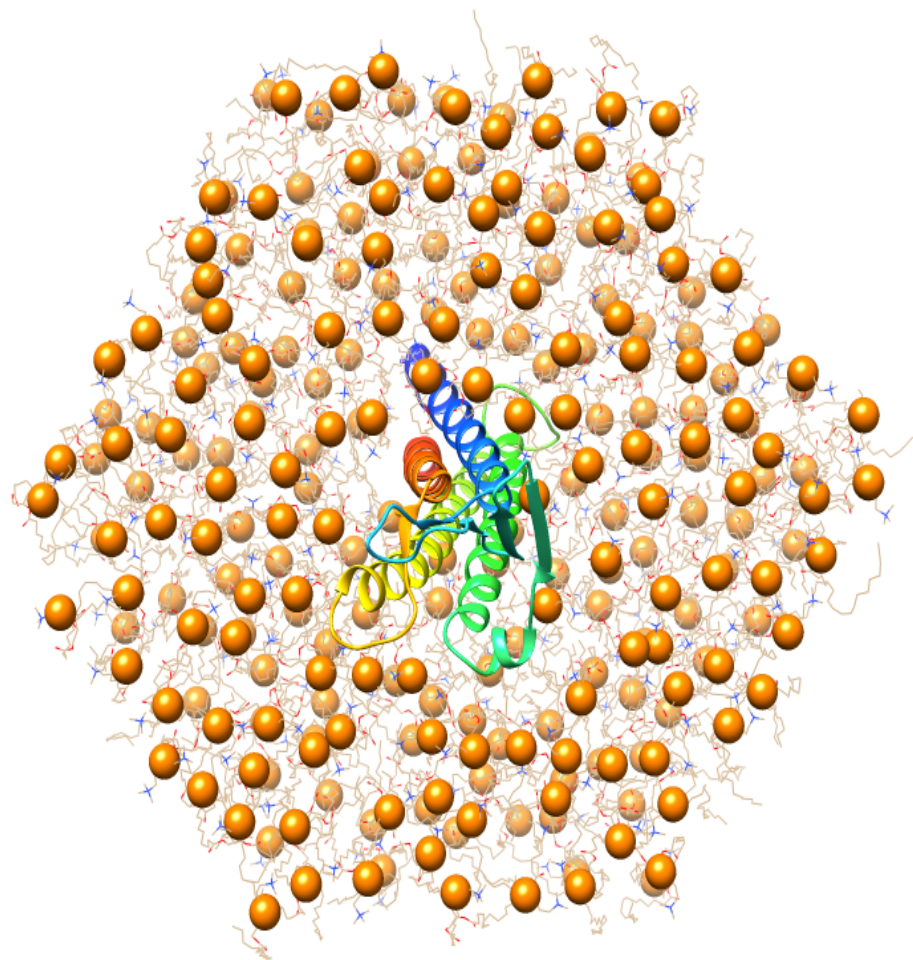

Supplement: S4 Fig — Viewed from the transmembrane domain (left) and from the extracellular environment (right). Cldn15 monomer (rainbow cartoon) is embedded in a POPC bilayer, shown as wire structures with sphere phosphorus atoms. Water molecules and ions are not shown for clarity. (PDF) [file pone.0184190.s005.pdf]

62 Å

62 Å

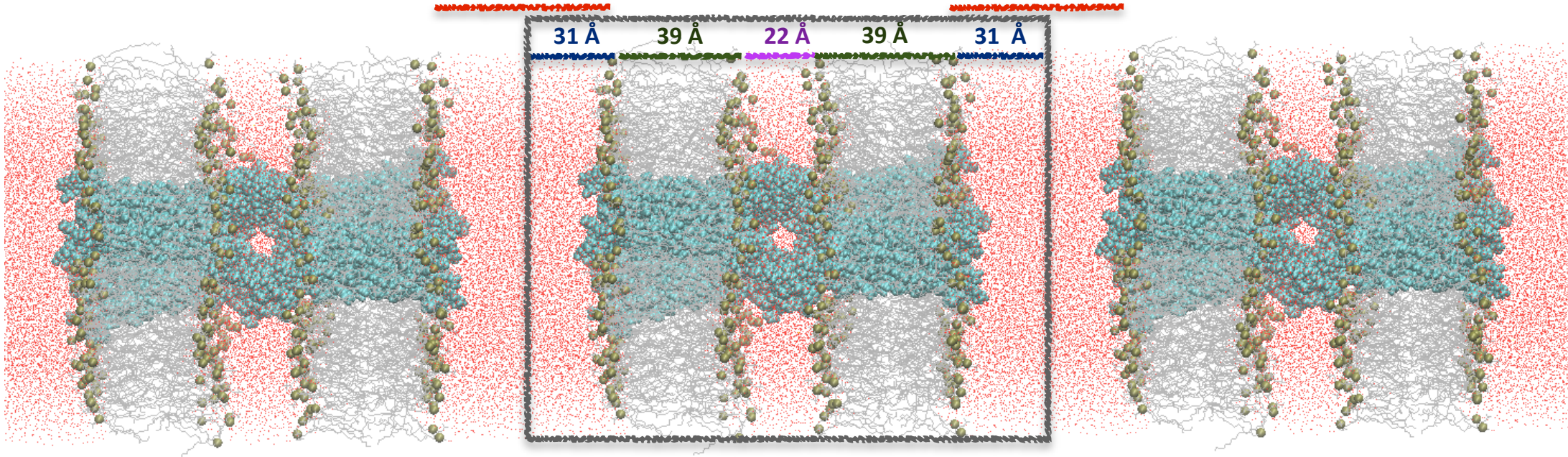

Supplement: S5 Fig — The unit box is indicated by gray lines and periodically repeated along z. Shown are POPC lipids in light gray (with phosphate atoms in brown), water (red), and the Cldn15 protomers of the channel in VDW style coloured cyan. Approximate dimensions for each compartment are indicated. (PDF) [file pone.0184190.s006.pdf]

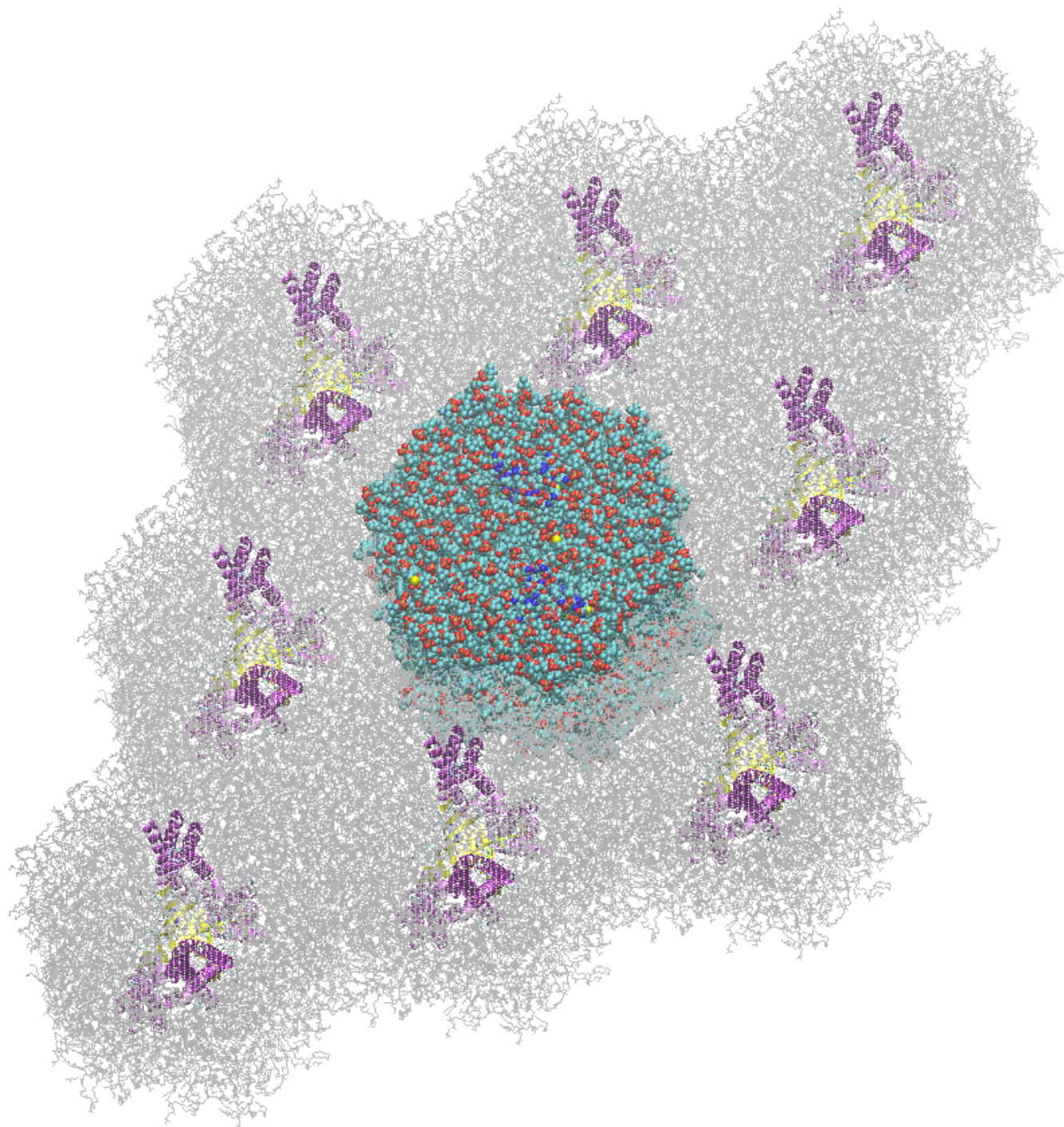

Supplement: S6 Fig — The unit box is shown as VDW spheres, while in the replicas lipids are pictured as gray lines and the the protein as ribbon with purple transmembrane domain and yellow extracellular region. Solvent molecules are not reported for clarity. (PDF) [file pone.0184190.s007.pdf]

**A**

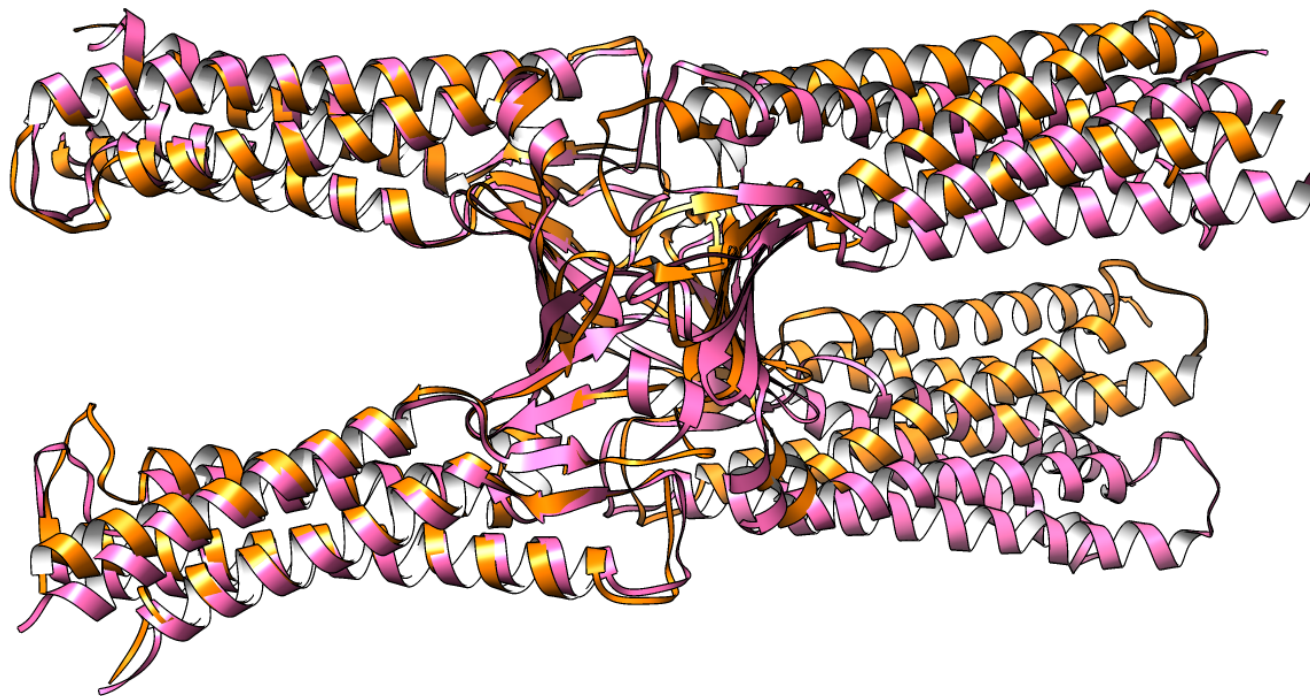

**B**

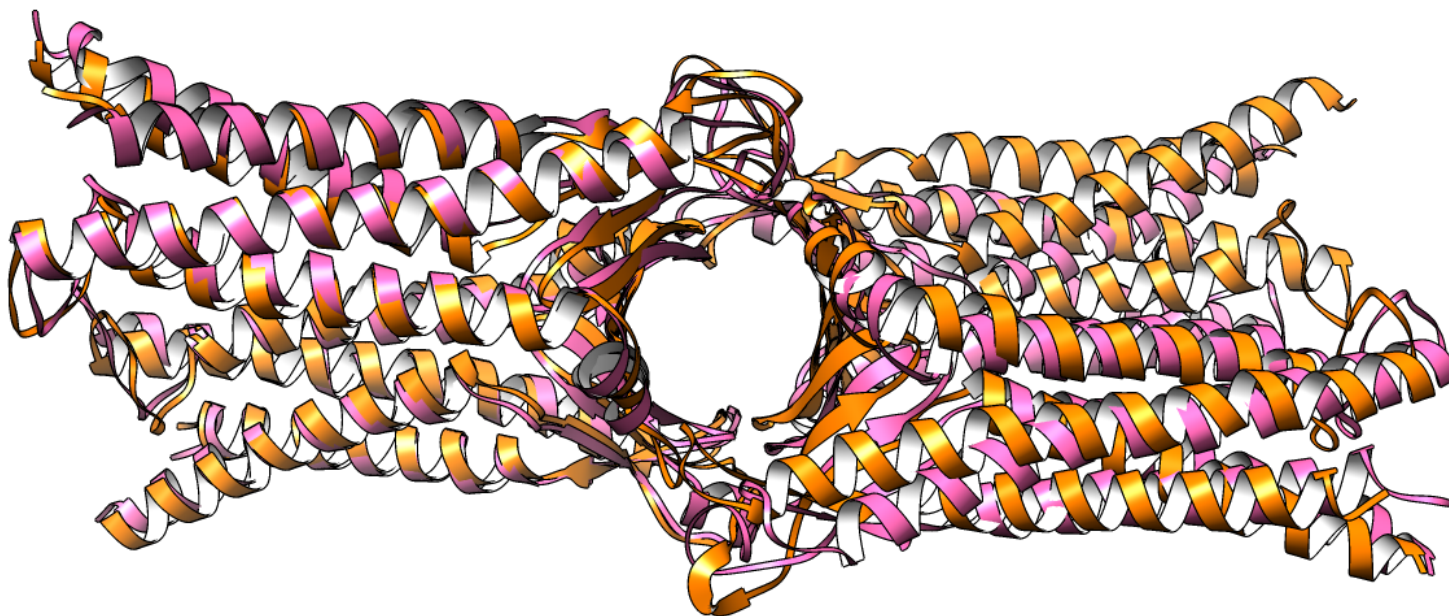

Supplement: S7 Fig — Superposition of the final configuration of the control simulation of the single pore structure (orange ribbons) and the structure taken from the main production run at the same time frame, ∼ 35 ns, (pink ribbons). A parallel with, and B perpendicular to, the elongation of the TJ strand. (PDF) [file pone.0184190.s008.pdf]

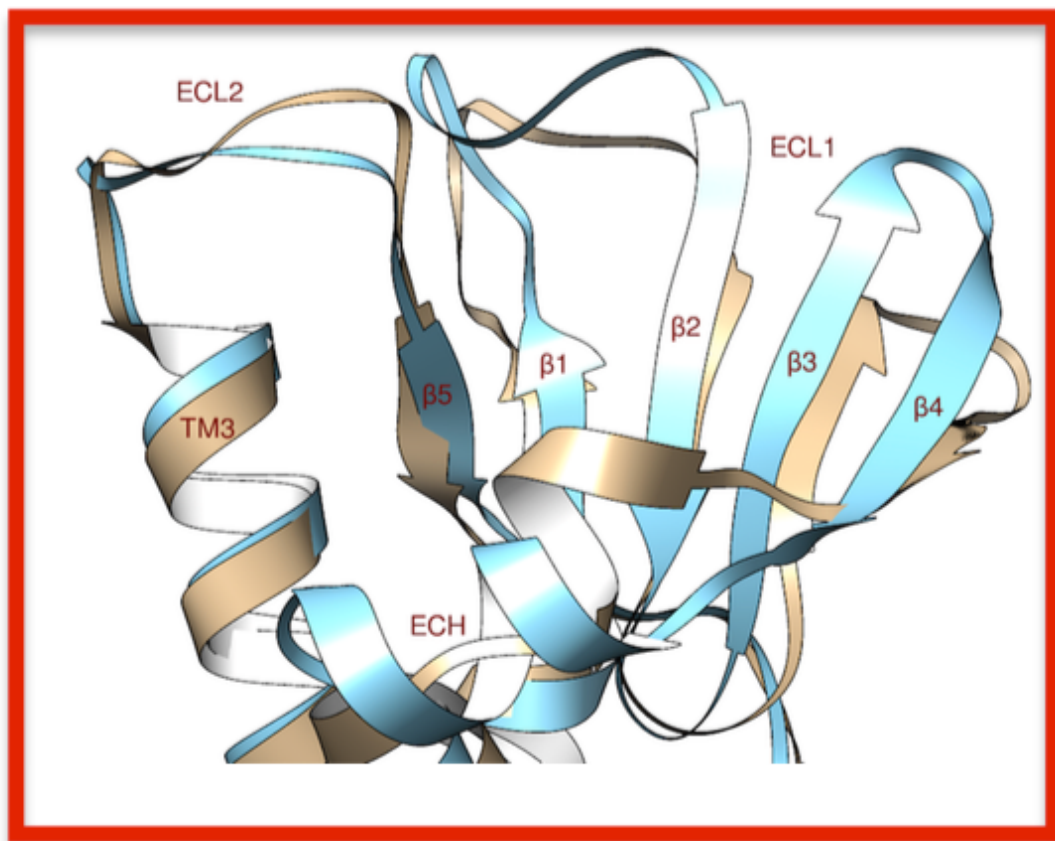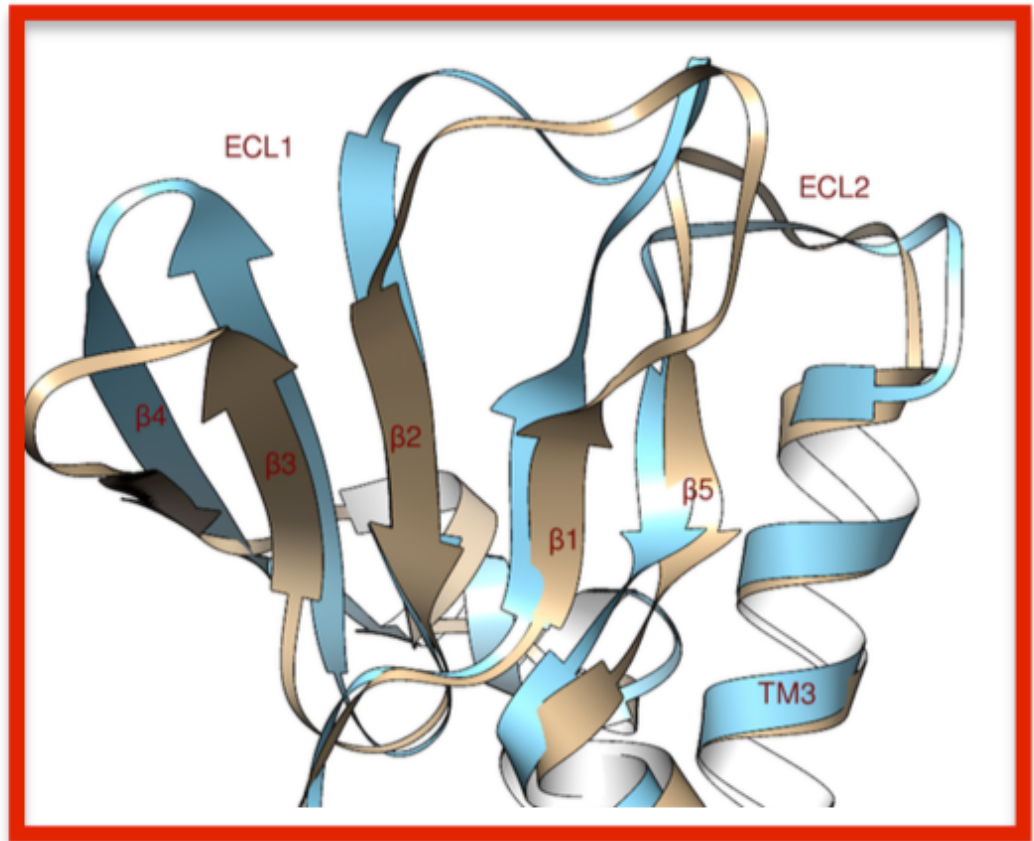

Supplement: S8 Fig — Superposition of the Model1 structure (cyan) and the final configuration from the MD run (brown) of Cldn15 monomer. (PDF) [file pone.0184190.s009.pdf]

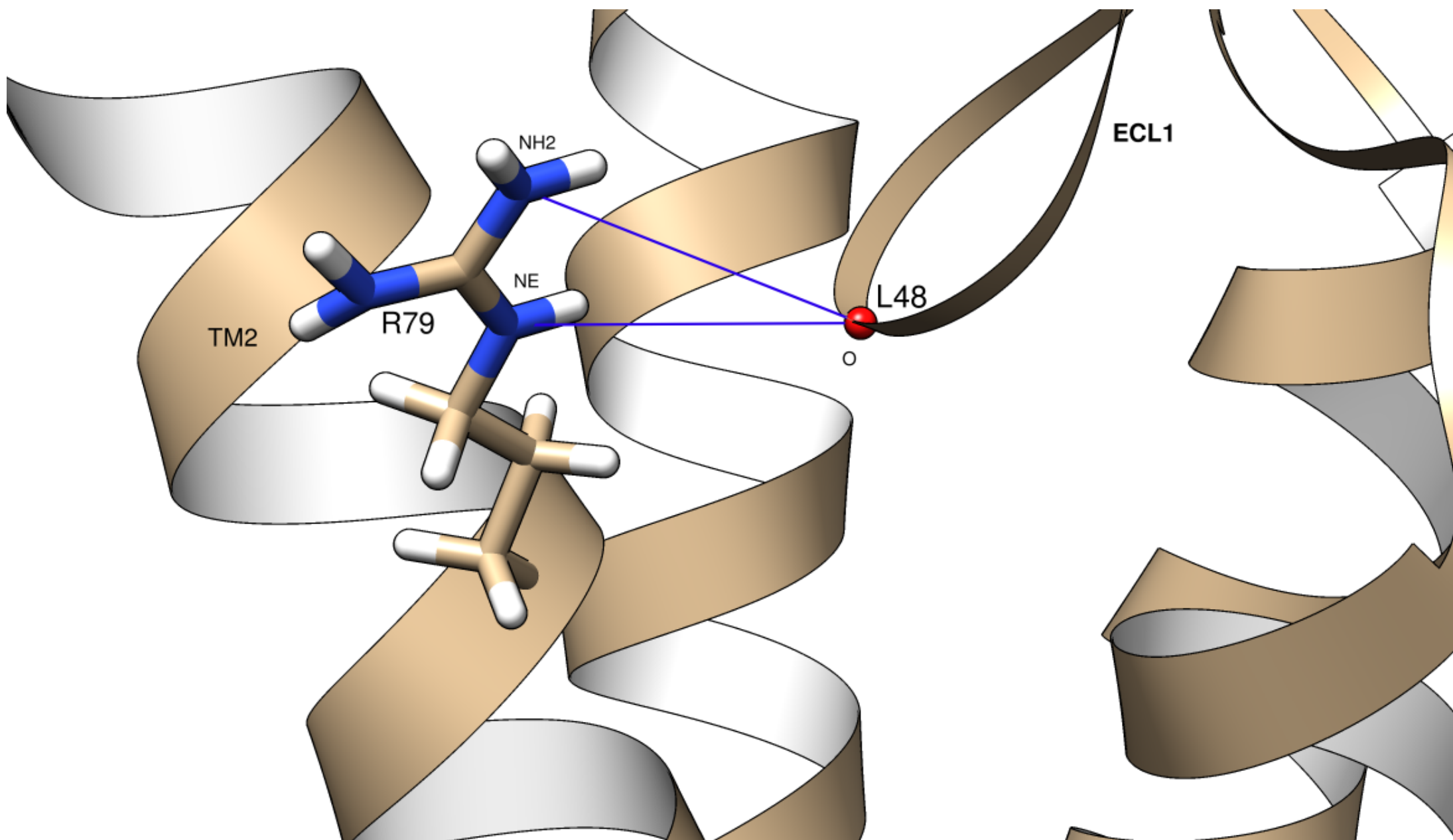

Supplement: S9 Fig — The side chain of R79 establishes two HBs with the main-chain carbonyl group of L48. (PDF) [file pone.0184190.s010.pdf]

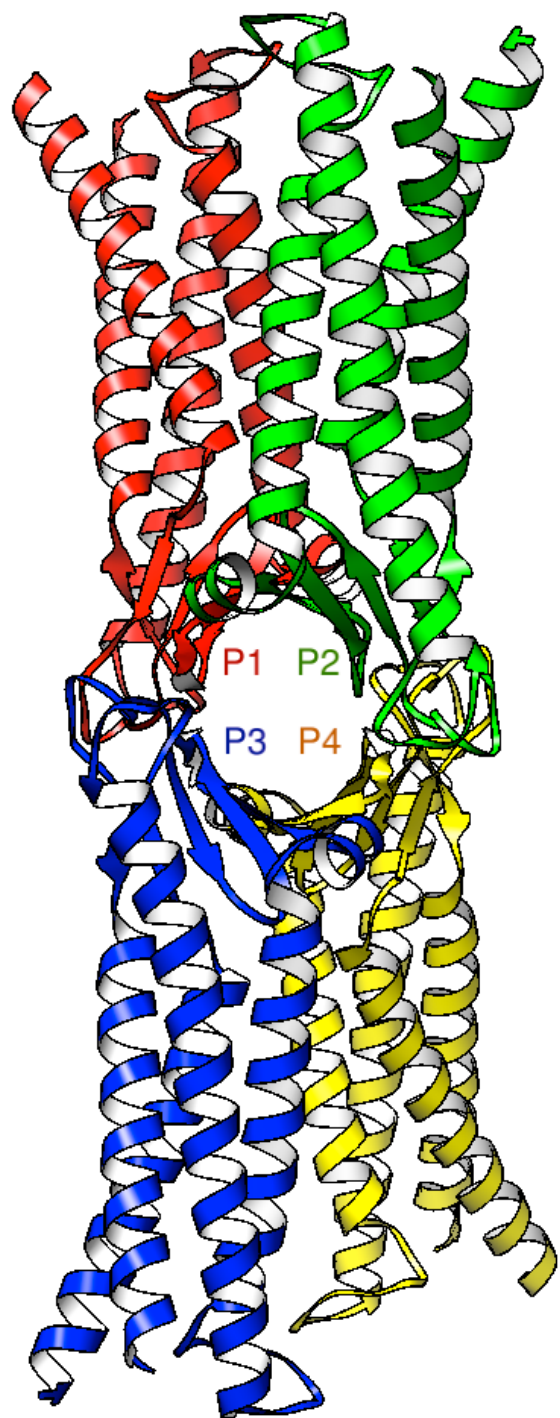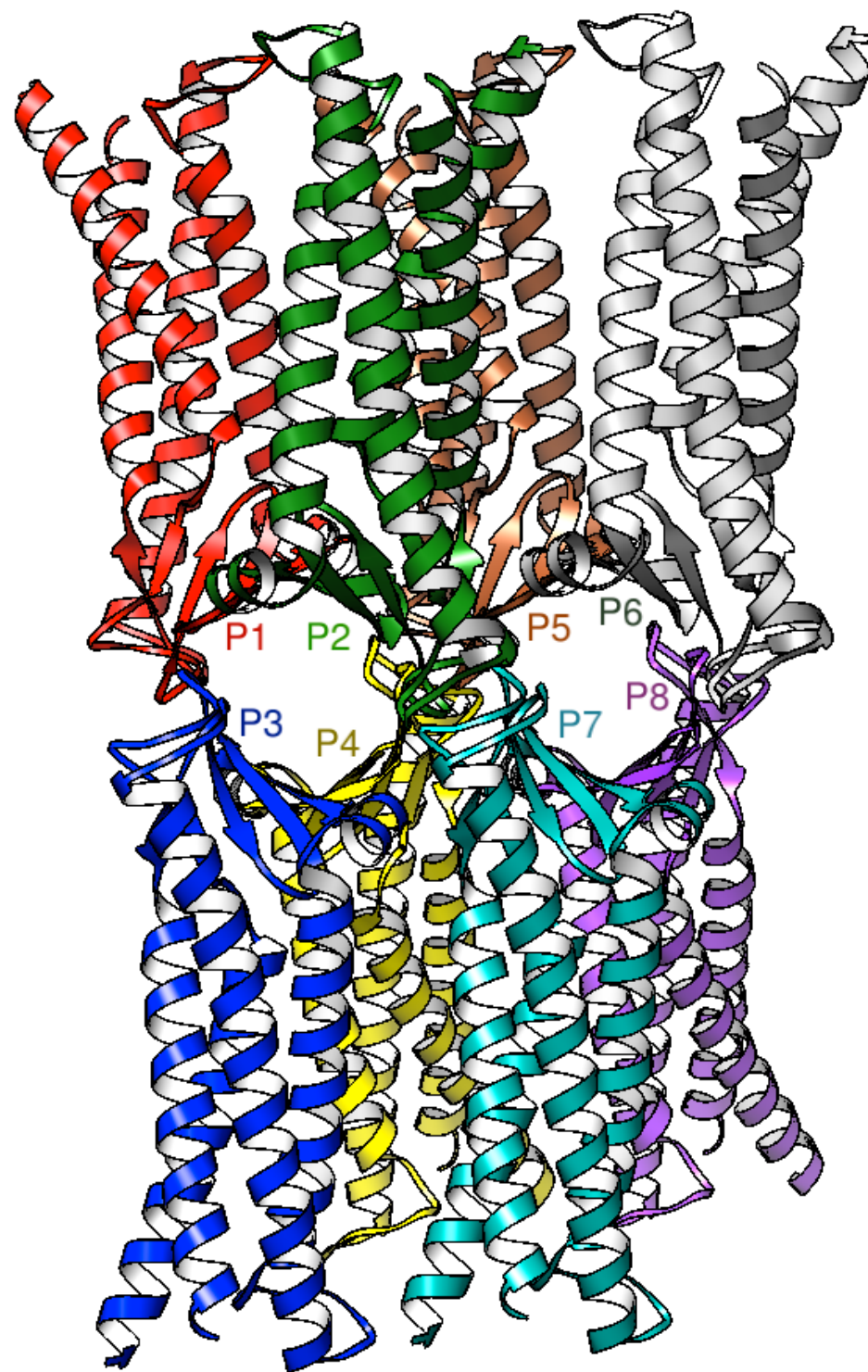

Supplement: S10 Fig — Ribbon representation of the single pore (left) and double pore (right) systems, with the labels of protomer segnames, used for the data analysis. (PDF) [file pone.0184190.s011.pdf]

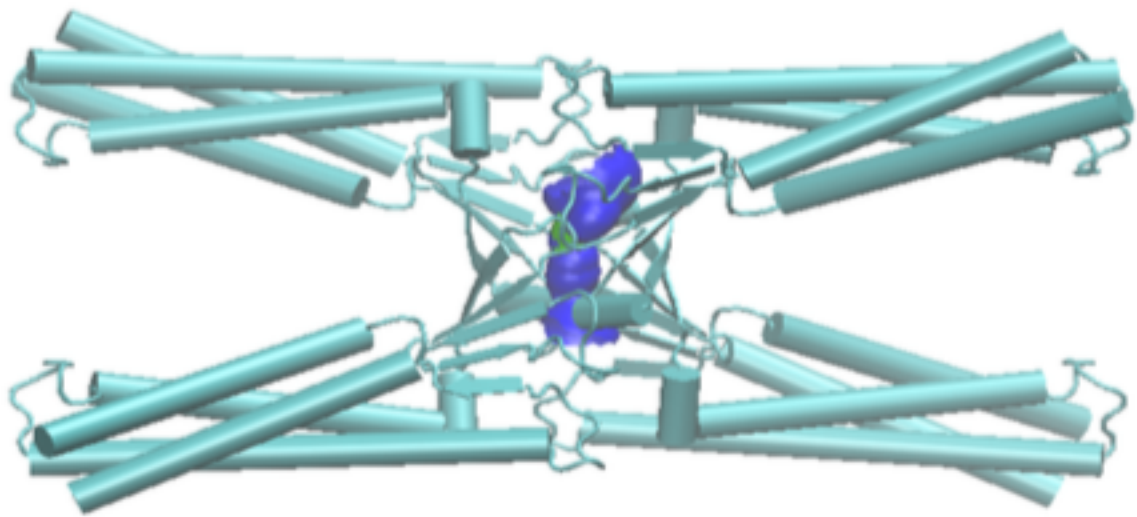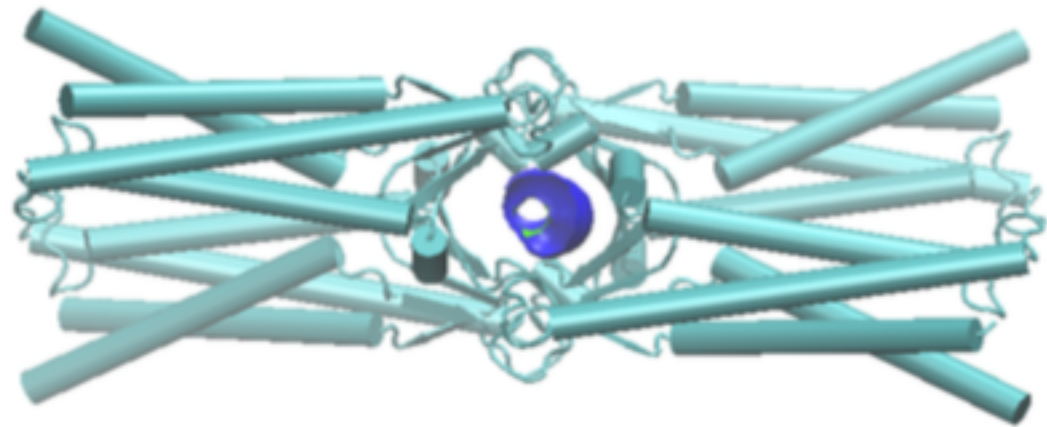

Supplement: S11 Fig — (PDF) [file pone.0184190.s012.pdf]

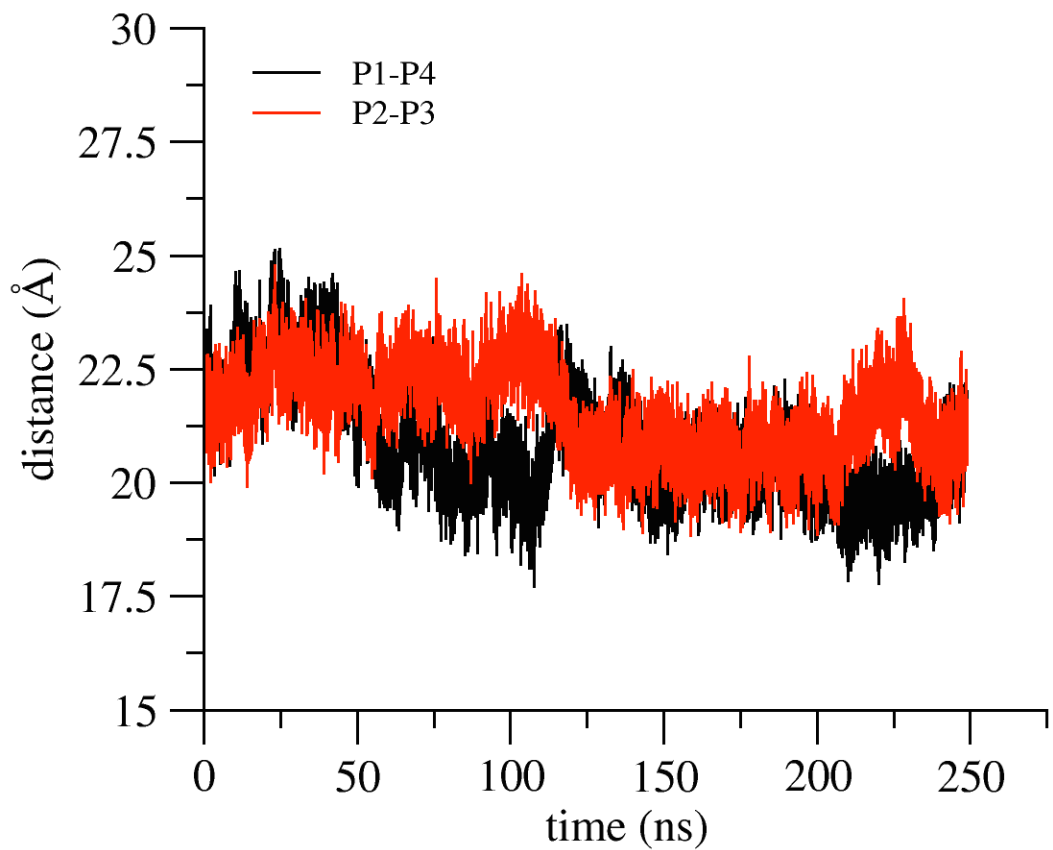

Supplement: S12 Fig — (PDF) [file pone.0184190.s013.pdf]

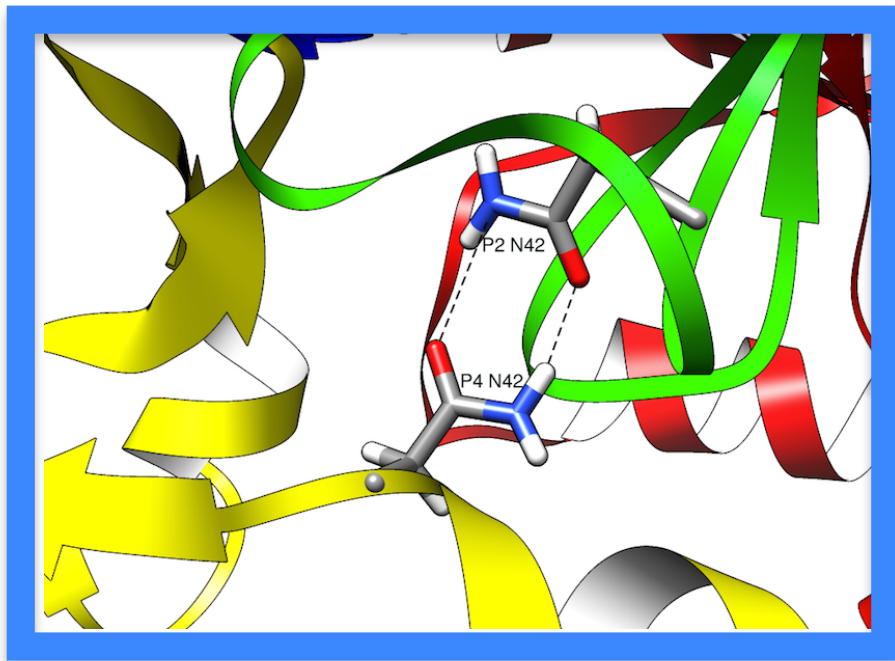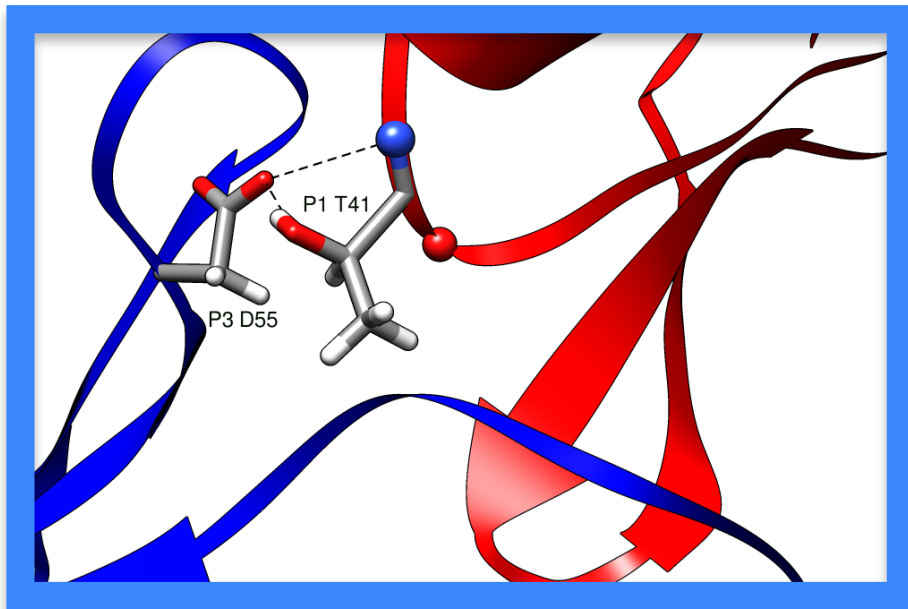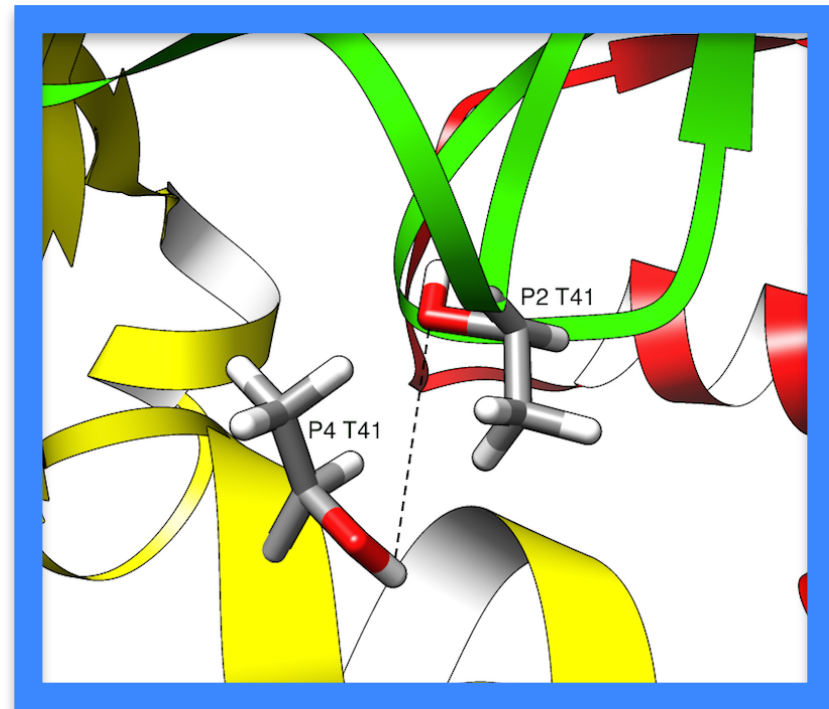

Supplement: S13 Fig — (PDF) [file pone.0184190.s014.pdf]

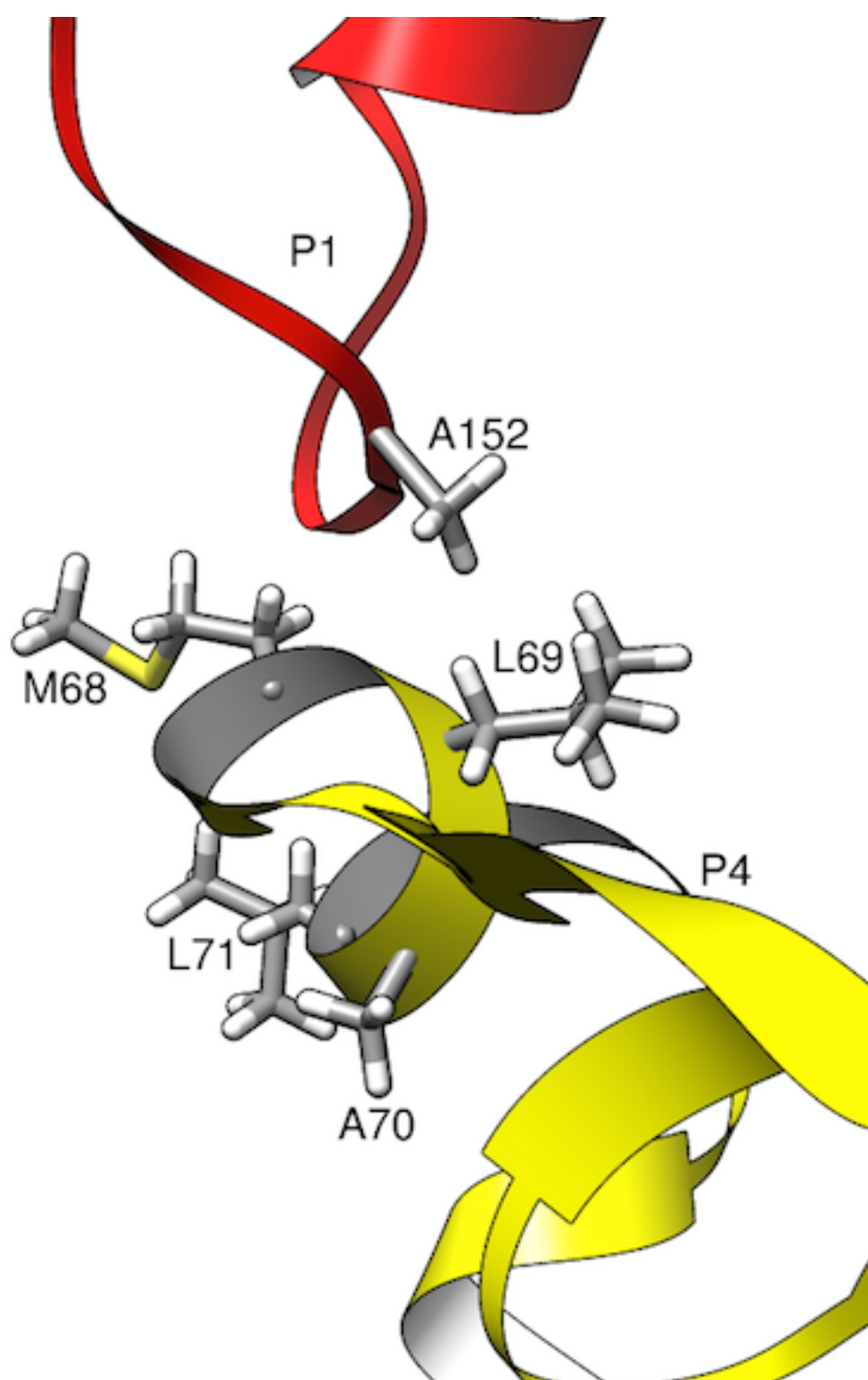

Supplement: S14 Fig — Contacts between the conserved residue A152 of P1 protomer and the conserved residues M68, L69, A70, L71 of the ECH region of P4 protomer. (PDF) [file pone.0184190.s015.pdf]

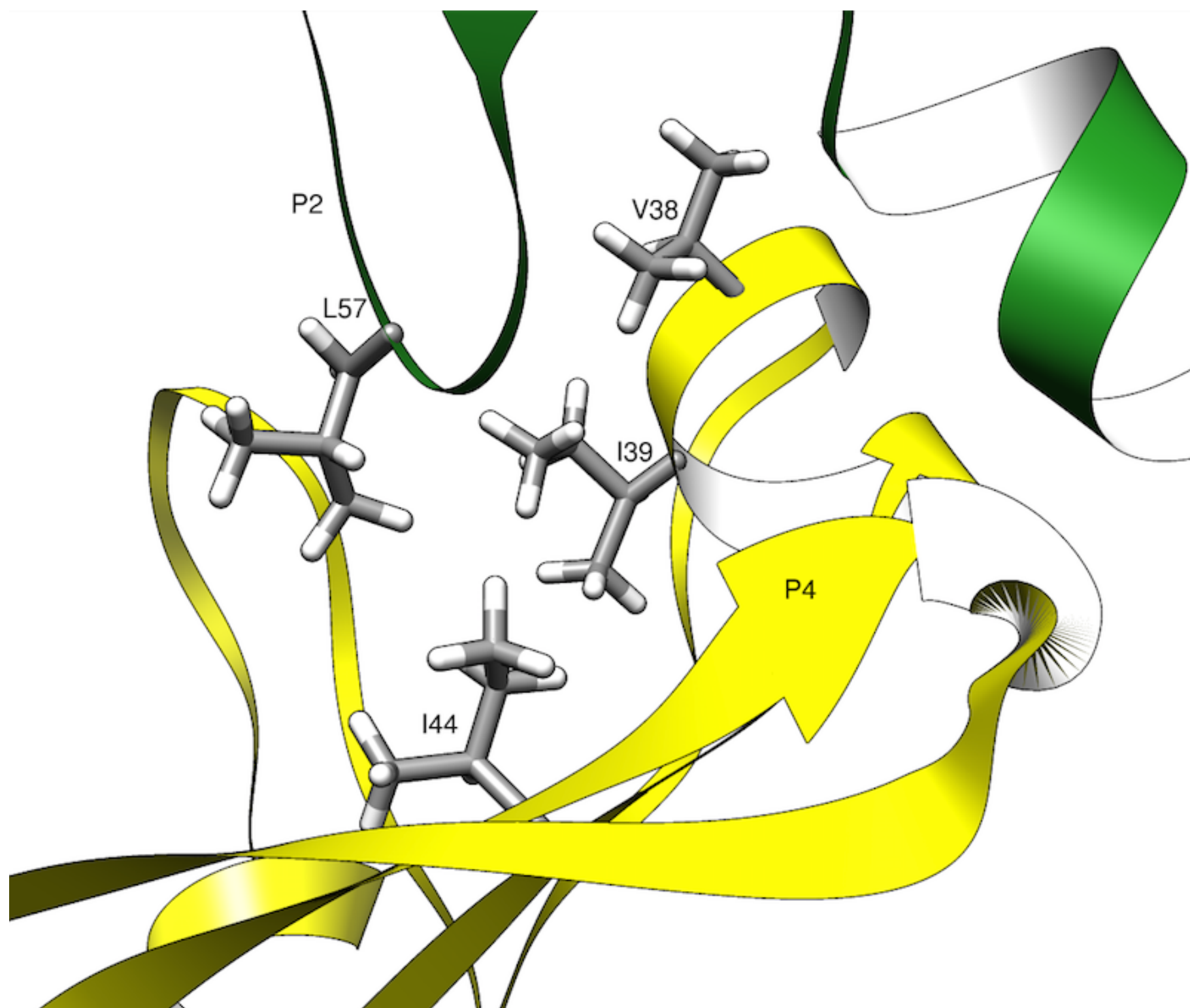

Supplement: S15 Fig — Hydrophobic interactions between ECL1 segments of diagonally opposed protomers. Specifically, L57 of P2 protomer is in close contact with the group of residues V38, I39 and I44 of P4 protomer. (PDF) [file pone.0184190.s016.pdf]

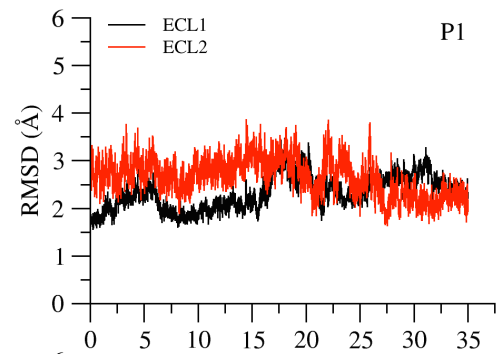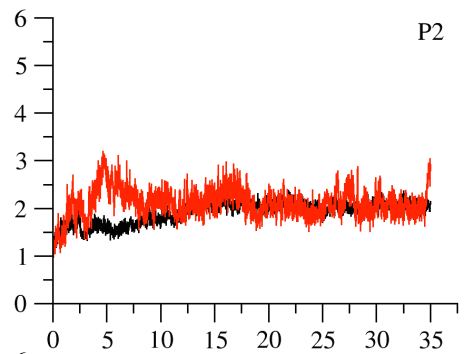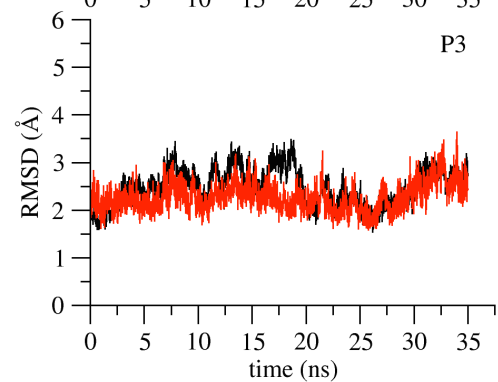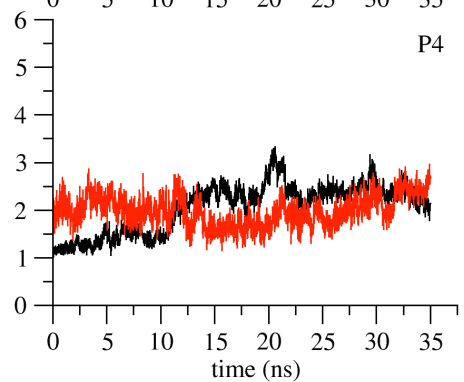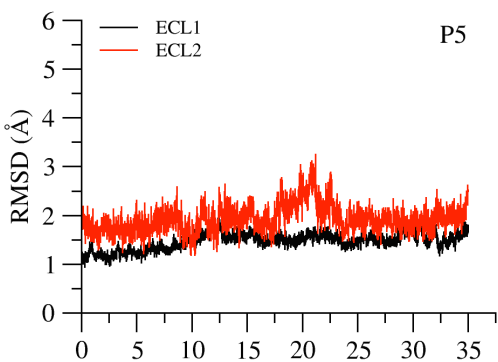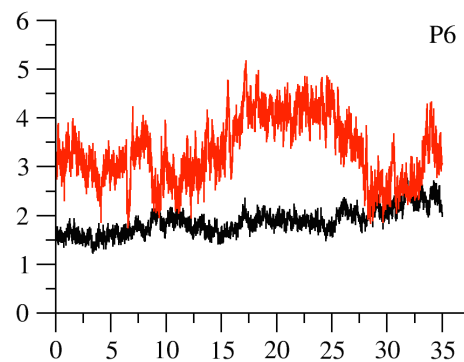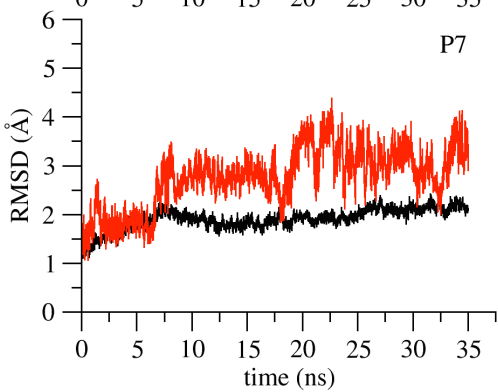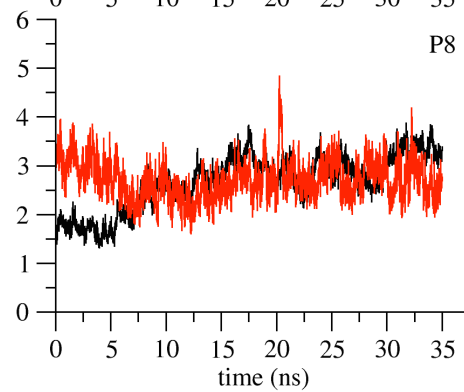

Supplement: S16 Fig — (PDF) [file pone.0184190.s017.pdf]

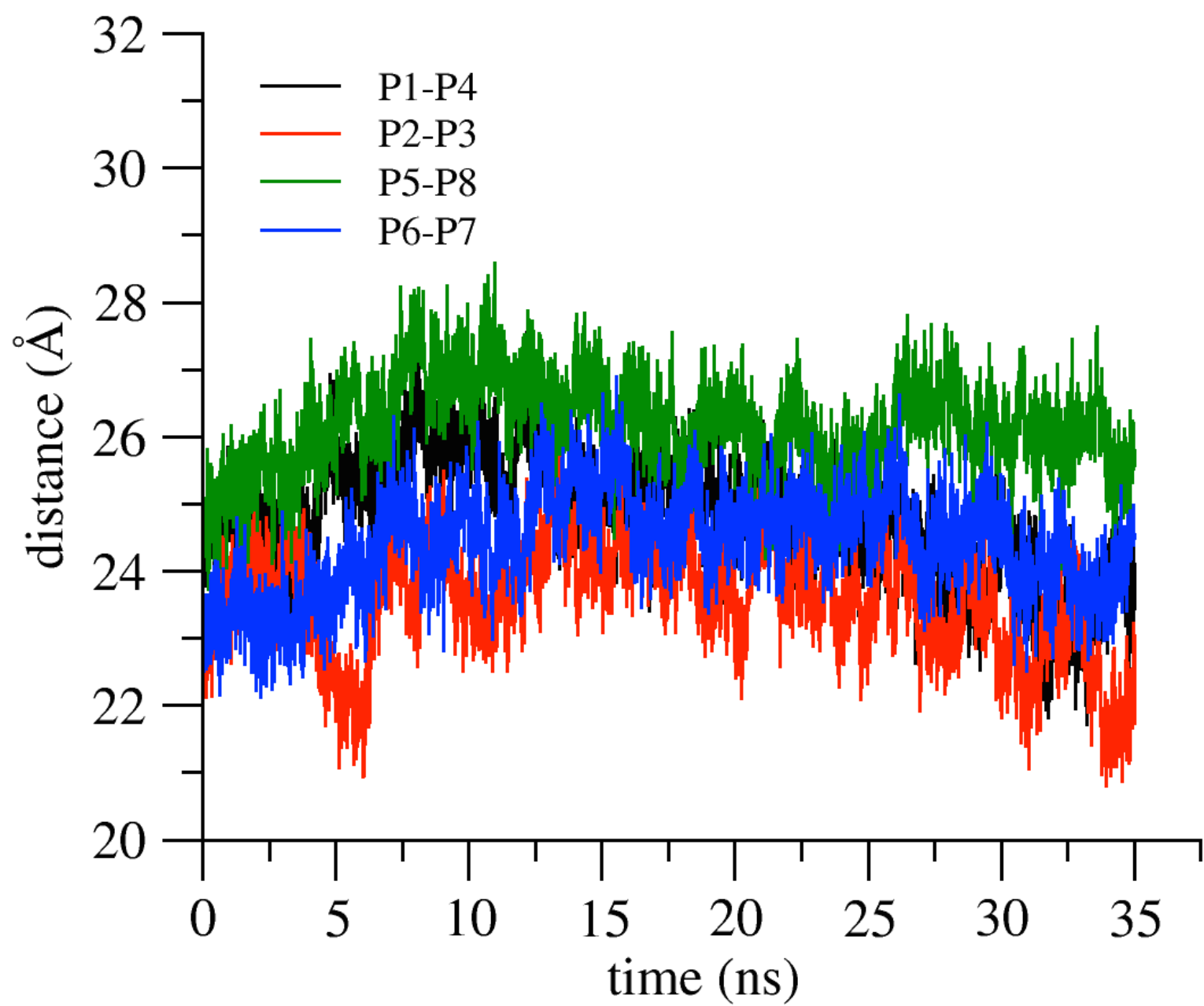

Supplement: S17 Fig — (PDF) [file pone.0184190.s018.pdf]
